# Dietary Polyphenols as Regulators of Protein Deposition and Muscle Health: Mechanisms, Interactions, and Functional Applications

**DOI:** 10.1002/fsn3.72487

**Published:** 2026-11-01

**Authors:** Chen Liu, Mengyi Ren, Xin Lai, Ke Gu, Liqiang Tan, Aimin Wu, Daiwen Chen

**Affiliations:** ^1^ Institute of Animal Nutrition Sichuan Agricultural University Chengdu Sichuan P. R. China; ^2^ College of Horticulture Sichuan Agricultural University Chengdu Sichuan P. R. China; ^3^ Sichuan Provincial Key Laboratory of Tea Resources Utilization and Quality Assessment Chengdu Sichuan P. R. China; ^4^ Institute of Subtropical Agriculture Chinese Academy of Sciences Changsha P. R. China; ^5^ Nutrition, Eumetabolism and Health Group Girona Biomedical Research Institute (IDIBGI‐CERCA) Salt Girona Spain

**Keywords:** human health, livestock production, muscle health, polyphenols, protein regulation, research models

## Abstract

Polyphenols are a class of natural compounds widely found in plants, attracting significant attention due to their antioxidant, anti‐inflammatory, and metabolic regulatory properties. In recent years, the role of polyphenols in regulating protein metabolism and deposition has gained considerable interest within the scientific community. This paper systematically reviews the mechanisms through which polyphenols influence protein synthesis, degradation, digestion, absorption, deposition, and muscle health. It highlights their regulation of protein synthesis via pathways such as mTOR/AMPK and PI3K/Akt signaling and their role in protein degradation through the ubiquitin‐proteasome system and the autophagy‐lysosomal system. Polyphenols also have significant effects on protein regulation and muscle health, such as promoting protein synthesis in skeletal muscle to support post‐exercise recovery and preventing muscle atrophy, regulating lipid metabolism, and aiding fat reduction in adipose tissue. The synergistic interaction of polyphenols with metabolic pathways involving amino acids, fats, and carbohydrates further enhances their comprehensive regulatory effect on protein metabolism. This paper also discusses the application prospects of polyphenols in animal husbandry and human health, including their potential value as feed additives and nutritional supplements. Future research should focus on elucidating the mechanisms of polyphenols across different species, food matrices, and processing conditions to facilitate their practical application in food science, animal nutrition, and human health.

## Graphical abstract

Dietary polyphenols contribute to the regulation of protein deposition and muscle health through multiple interconnected mechanisms. They promote protein synthesis and myogenic differentiation mainly by modulating the PI3K/AKT/mTOR signaling pathway and myogenic regulatory factors, while suppressing excessive protein degradation through regulation of the ubiquitin‐proteasome and autophagy‐lysosomal systems. Polyphenols also improve muscle energy metabolism by enhancing glucose uptake and fatty acid oxidation through AMPK/AKT‐related pathways. Collectively, these coordinated effects support muscle protein homeostasis and provide a mechanistic basis for the potential application of dietary polyphenols in animal husbandry, food manufacturing, and functional nutritional supplements.

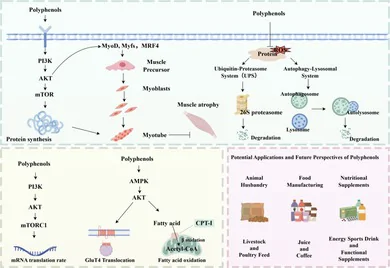

## 1 Introduction

Physiological protein deposition refers to the net accumulation of protein within tissues when protein synthesis exceeds degradation. Unlike pathological protein deposition, which can lead to harmful protein buildup associated with neurodegenerative diseases, physiological protein deposition supports tissue structure, repair, and functional adaptability, particularly in muscle and bone (Aggarwal and Bains 2022). In muscle tissue, protein deposition is essential for maintaining muscle mass, strength, and physical performance (Nunes et al. 2022). With aging, muscle loss and osteoporosis often occur, reducing physical capability and quality of life (Dominique et al. 2021). Studies have shown that reduced protein deposition in elderly individuals contributes to the weakening of skeletal and muscular tissues, increasing the risk of falls and fractures (Weaver et al. 2021). Adequate protein intake and exercise are therefore central to strategies for preserving lean mass and supporting healthy aging.

Beyond its importance for human health, protein deposition is also crucial in agriculture and livestock production, directly affecting animal growth rates and meat quality (Son et al. 2025). Research into the regulation of protein deposition offers theoretical and practical support for improving feed conversion efficiency and growth outcomes. Optimizing protein deposition in muscle tissue has been shown to significantly improve the texture and nutritional profile of meat, as demonstrated in studies with *Eriocheir sinensis* (Zhang, Chi, et al. 2021). For example, by balancing amino acids in animal feed, protein can be converted more effectively into muscle tissue, resulting in improved tenderness and nutrient density (Li et al. 2025). Additionally, the inclusion of specific polyphenols or antioxidants in feed has been shown to reduce protein oxidation, thereby extending meat shelf life and enhancing flavor, quality, and food safety (Huang et al. 2022; Nuerjiang et al. 2023). Understanding how dietary factors regulate protein turnover is therefore relevant to both healthy aging and efficient, sustainable animal production (Su et al. 2022).

Polyphenols are involved in regulating protein metabolism, making them important modulators of protein deposition and muscle protein synthesis (Nikawa et al. 2021; Nuerjiang et al. 2023; Salucci and Falcieri 2020; Xiang et al. 2023). They are a diverse group of naturally occurring secondary metabolites found abundantly in plants, including classes such as flavonoids, phenolic acids, stilbenes, anthocyanins, and tannins (Huang et al. 2022). Their basic chemical structure consists of multiple phenolic rings, which contribute to their ability to interact with various cellular targets (Nuerjiang et al. 2023). This structural diversity underlies their wide range of biological activities, including antioxidant, anti‐inflammatory, and metabolic regulation (Huang et al. 2022). Primary sources of polyphenols include tea (rich in catechins), coffee (containing chlorogenic acids), grapes (high in resveratrol), berries (rich in anthocyanins), and vegetables (containing phenolic acids) (Nuerjiang et al. 2023). Experimental studies indicate that selected polyphenols can influence muscle‐related protein turnover in a compound‐ and model‐dependent manner. Quercetin promoted Akt/mTORC1/4E‐BP1 signaling and myogenic differentiation in C2C12 cells (Hour et al. 2022), whereas dietary quercetin increased breast‐ and thigh‐muscle protein contents in broilers (Xiao et al. 2021). Curcuminoids and EGCG have also reduced MuRF1‐ and Atrogin‐1‐associated proteolytic responses in experimental muscle‐atrophy models (Chang et al. 2020; Sani et al. 2021).

Polyphenols regulate protein metabolism by modulating key signaling pathways, such as mTOR (mammalian target of rapamycin) and AMPK (adenosine monophosphate‐activated protein kinase) (Herrera‐Balandrano et al. 2021), which are critical for protein synthesis and energy balance (Langer et al. 2024; Sartori et al. 2021). Additionally, polyphenols help regulate oxidative stress, which supports cellular health by reducing oxidative damage to proteins and cellular components, thereby maintaining the cellular environment needed for efficient protein synthesis. Through these mechanisms, polyphenols may enhance anabolic processes in muscle tissue, reduce protein degradation, and facilitate cellular repair and regeneration, though more research is needed to confirm these effects across different contexts and species (Nikawa et al. 2021; Nuerjiang et al. 2023; Nunes et al. 2024; Yue et al. 2021).

Several recent reviews have examined the relationship between polyphenols and skeletal muscle from different perspectives. Earlier reviews focused primarily on the anti‐atrophic effects of polyphenols under catabolic or pathological conditions (Nikawa et al. 2021; Salucci and Falcieri 2020), whereas subsequent work considered a broader range of dietary plant extracts in relation to skeletal muscle development and metabolic function (Xiang et al. 2023). Medoro et al. (2024) further synthesized clinical evidence regarding polyphenol supplementation in individuals with sarcopenia. More recent reviews have examined protein‐ and polyphenol‐based nutritional strategies for age‐related sarcopenia (Aragón‐Espinosa et al. 2026) and the molecular effects of polyphenols on skeletal muscle development and homeostasis (Mattioli et al. 2026). Collectively, these studies highlight the potential of polyphenols in maintaining muscle mass and function. However, they are largely centered on muscle atrophy, sarcopenia, or human health applications, and do not integrate evidence on physiological protein turnover and net protein deposition across both human and livestock systems.

Accordingly, this review extends previous work by shifting the focus from muscle atrophy alone to overall protein turnover and net deposition, and by integrating evidence across molecular mechanisms, metabolic regulation, and multiple experimental models. It further links mechanistic insights to both human muscle health and livestock production outcomes, while also highlighting inconsistencies and gaps that limit current translation.

## 2 Direct Regulation of Protein Turnover by Polyphenols

### 2.1 Role of Polyphenols in the Regulation of Protein Synthesis

Protein synthesis is coordinated principally by nutrient‐, energy‐, and growth‐factor‐sensitive signaling. Activation of the insulin‐like growth factor 1 (IGF‐1)/phosphoinositide 3‐kinase (PI3K)/Akt pathway stimulates mechanistic target of rapamycin complex 1 (mTORC1). Activated mTORC1 promotes translation by phosphorylating ribosomal protein S6 kinase 1 (S6K1) and 4E‐binding protein 1 (4E‐BP1), thereby relieving the inhibitory effect of 4E‐BP1 on eIF4E. Conversely, under energy stress, AMP‐activated protein kinase (AMPK) restrains mTORC1 through TSC2 and Raptor and promotes ULK1‐dependent autophagy (Goul et al. 2023; Steinberg and Hardie 2023; Yang et al. 2022). Importantly, these pathways should not be interpreted as uniformly anabolic or catabolic, because their consequences depend on the cell type, physiological or pathological state, compound, dose, and duration of exposure. In A549 non‐small‐cell lung cancer cells, resveratrol activated AMPK and suppressed mTOR, thereby promoting autophagy and apoptosis; by contrast, quercetin activated Akt/mTOR/4E‐BP1 signaling in C2C12 myoblasts and promoted protein synthesis and myogenic differentiation (Hour et al. 2022; Li et al. 2022). Thus, inhibition of anabolic signaling may be beneficial in rapidly proliferating cancer cells, whereas activation of the same pathway may support muscle development, indicating that polyphenol effects should not be extrapolated across tissues or disease states.

Animal studies provide evidence closer to whole‐body protein deposition. Dietary supplementation with 0.2 g/kg quercetin increased protein content in the breast and thigh muscles of broilers and altered IGF‐1/PI3K/Akt/mTOR‐associated translational regulators (Xiao et al. 2021). Curcumin has similarly been associated with preservation of myotube size or muscle mass under catabolic conditions, together with changes in IGF‐1/Akt/mTOR signaling and oxidative or inflammatory status (Lee et al. 2021; Nunes et al. 2024; Rauofi et al. 2020; Sani et al. 2021). However, increases in tissue protein content, muscle mass, or anabolic‐pathway phosphorylation should not automatically be interpreted as direct increases in protein synthesis. Most of these studies did not measure fractional protein synthesis rates using tracer‐based methods, and some of the observed preservation of muscle mass may instead reflect reduced protein degradation. Representative cell and animal studies are summarized in Tables 1 and 2, and the principal direct signaling mechanisms are illustrated in Figure 1.

**TABLE 1:** In vitro cell models for studying the positive effects of dietary polyphenols on protein regulation and potential muscle health.

Cell type	Modeling agent/method	Polyphenol and dosage	Experimental duration	Effect	Key mechanism	References
C2C12 (mouse skeletal muscle precursor cell line)	H_2_O_2_ (0.25 mM)	Licochalcone B (5–20 μM)	36 h	Alleviated oxidative damage and reversed the dysregulation of antioxidant and myogenic genes	Inhibiting the expression of antioxidant enzyme‐related genes (Nqo1, Hmox1, Cat, Sod1, Gpx1), enhancing the expression of multiple myosin heavy chain genes (Myh2, Myh4, Myh7)	(Kim et al. 2025)
	\	Quercetin (12.5 μM)	7 days	Stimulated myoblast migration and myogenic differentiation	Activating the STAT3 and AKT signaling pathways promotes myogenic differentiation, modulates the ITGB1 signaling pathway, and induces phosphorylation of FAK and paxillin	(Hour et al. 2022)
	TNF‐α (40 ng/mL)	Formononetin (5 and 50 μmol/L)	48 h	Anti‐inflammatory, improved the dysfunction of satellite cell proliferation and differentiation	Inhibiting MuRF‐1, MAFbx, and myostatin expression, and increasing phosphorylation of PI3K, Akt, and FoxO3a	(Liu et al. 2021)
	TNF‐α (100 ng/mL)	Resveratrol (100 μM)	24 h	Induced hypertrophy in C2C12 myotubes and inhibited atrophy responses	Inducing activation of the Akt/mTOR pathway, reducing FoxO1 protein, and promoting FoxO1 phosphorylation	(Wang, Yin, et al. 2022)
	Cisplatin (40 μM)	Daidzein (25 μM)	24 h	Inhibited protein degradation and alleviated myotube atrophy	Inhibiting the Glut4/AMPK/FoxO pathway to reduce myotube atrophy	(Zhang, Chi, et al. 2021)
	C26 conditioned culture medium	Magnolol (10 or 20 μM)	Changed the medium every 48 h and collect on day 5	Increased protein synthesis to inhibit myotube atrophy	Upregulating the expression levels of MyHC, MyoD, and MyoG, increasing the phosphorylation of mTOR, AKT, and P70S6K, and reducing the expression of ubiquitin, Atrogin‐1, and MuRF‐1	(Ge et al. 2020)
	H_2_O_2_ (100 μM)	Tannase‐converted green tea extract (EC, EGC and GA: 1–20 μg/mL)	48 h	Increased myotube density	Upregulating myogenin, Myf5, and MyoD mRNA expression, increasing mTOR protein levels and elevating GST mRNA levels	(Hong et al. 2020)
	Advanced glycosylation end‐products (100 μg/mL)	Highland barley tea polyphenols (20–200 μg/mL)	24 h	Suppressed muscle atrophy and myogenesis signaling pathways	Reducing MDA content, upregulating expression of SIRT3 and Myh proteins, and inhibiting Atrogin‐1 expression	(Nie et al. 2022)
	\	EGCG (50 μM)	24 h	Promoted myoblast differentiation	Upregulating miR‐486 expression, stimulateing AKT phosphorylation and inhibiting FoxO1a‐mediated transcription of MuRF1 and Atrogin‐1	(Chang et al. 2020)
	Dexamethasone (10 μM)	Trifuhalol A (2.5, 5, 10 μg/mL)	2, 4, and 6 days	Promoted protein synthesis and alleviated muscle atrophy and degeneration	Downregulating the mRNA levels of atrophy‐related genes (FoxO3a, MAFbx/MuRF‐1) and upregulating the myogenic factor MyoD and its key regulatory proteins (p‐Akt, p‐mTOR, MyHC)	(Yang et al. 2025)
	Dexamethasone (1 μM)	EGCG (5 μM) Combined EGCG (2.5 μM) and eriodictyol (2.5 μM)	24 h	Prevented atrophy in C2C12 myotubes	Downregulating FoxO3a expression to inhibit MuRF1 expression and suppressing MuRF1 expression through 67LR Suppressing the expression of MuRF1, with eriodictyol enhancing the inhibitory effect of EGCG on MuRF1	(Murata et al. 2020)
	Dexamethasone (250 μM)	Quercetin (25, 50 or 100 μM)	28 h	Inhibited apoptosis	Reducing Akt dephosphorylation by decreasing atrogin‐1 expression, upregulating Bcl‐2 expression, and reducing caspase‐3/9 activity	(Chen et al. 2020)
	Dexamethasone (10 μM)	Morin (10 μM)	25 h	Reduced oxidative stress and inhibited protein degradation	Inhibiting ROS accumulation, Foxo3a expression, and the expression of MAFbx1/atrogin‐1, MuRF‐1, and Cbl‐b	(Ulla et al. 2021)
Human skeletal muscle cells	Cisplatin (20 μM)	Oligonol	48 h	Inhibited protein degradation	Suppressing the NF‐κB/MuRE1 axis and activating the Akt/mTOR/p70S6K pathway	(Chang et al. 2025)

**TABLE 2:** Animal models for studying the effects of dietary polyphenols on protein regulation and muscle health.

Animal	Modeling agent/method	Polyphenol and dosage	Experimental duration	Effect	Key mechanism	References
BALB/c mice	Fatigue motion	*Lonicera caerulea* berry polyphenols extract (250 mg/kg bw/d)	6 weeks	Inhibited apoptosis of skeletal muscle cells, promoted cell proliferation, and reduced muscle damage	Activating the miRNA‐133a/IGF‐1/PI3K/Akt/mTOR pathway promotes the proliferation of skeletal muscle cells	(Liu, Meng, et al. 2022)
ICR mice	Dexamethasone (1 mg/kg bw/d)	*Curcuma longa* L. water extract (1 g/kg bw/d)	Daily oral gavage for 3 consecutive weeks	Enhanced antioxidant capacity, prevented muscle atrophy	Enhancing antioxidant enzyme activity and increasing the expression levels of MuRF‐1 and Atrogin‐1	(Kim et al. 2021)
	Weight‐loaded forced swimming test	Licochalcone B (5, 50 and 500 mg/kg/d)	Daily oral administration for 28 days	Ameliorated oxidative stress, enhanced mitochondrial biogenesisand alleviated skeletal muscle fatigue	Upregulating the TIGAR/SIRT1/PGC‐1α signaling pathway to enhance the expression of SOD2, CAT, and GPx1, reducing ROS levels	(Kim et al. 2025)
Sprague–Dawley (SD) rats	Nephrectomy	Formononetin (30 and 60 mg/kg/d)	Daily oral gavage for 6 weeks	Anti‐inflammatory, alleviated myostatin‐mediated muscle atrophy	Inhibition of MAFbx, MuRF‐1, and myostatin expression, and upregulation of phosphorylation in the PI3K, Akt, and FoxO3a pathways	(Liu et al. 2021)
	Resistance training	Chlorogenic acid (50 or 100 mg/kg)	Daily gavage administration for 56 days	Improved muscle function	Increasing muscle mitochondrial ATP levels and enhancing mitochondrial dehydrogenase activity	(Ommati et al. 2020)
	\	Curcumin (10, 50 mg/kg) and gamma‐oryzanol (10 mg/kg) solid dispersions	Daily oral gavage for 6 consecutive weeks	Enhanced antioxidant capacity, ameliorated age‐related muscle atrophy, and reduced muscle damage	Activating Nrf2 pathway to enhances antioxidant enzyme activity, reducing ubiquitin‐proteasome‐mediated protein degradation, and upregulating IGF‐1/Insulin‐Akt–mTOR signaling as well as GLUT4 expression	(Taengthong et al. 2022)
SAMP8 mice	\	Normal diet containing 0.5% catechins	Daily feeding for of 12 weeks	Improvedage‐related mitochondrial capacity and promoted myoblast differentiation.	Increasing the CSA of both Type I and II muscle fibers and regulating the expression of MyoD	(Matsuzaki et al. 2025)
	\	Chow diet containing 0.32% EGCG	Daily feeding for of 8 weeks	Alleviated age‐related muscle loss	Upregulation of miR‐486 expression, reducing PTEN protein levels, enhancing AKT phosphorylation, and downregulating the expression of MuRF1 and Atrogin‐1	(Chang et al. 2020)
	Exercise training	Resveratrol (150 mg/kg bw)	Oral administration twice a week for 1 month	Ameliorated apoptosis and alleviated sarcopenia	Mitigating age‐related mitochondrial dysfunction by reducing the expression of Bad, caspase‐3, and interleukin‐6, decreasing the activity of FGF2 and UPA in skeletal muscle	(Bai et al. 2020)
LLC tumor‐bearing mice	Cisplatin (4 mg/kg/3d, i.p.)	Daidzein (20 or 80 mg/kg/d)	Oral administration once daily for 12 consecutive days	Inhibited protein degradation and alleviated skeletal muscle atrophy	Inhibiting the Glut4/AMPK/FoxO pathway, downregulating the expression of Atrogin1 and MuRF1, and suppressing skeletal muscle protein degradation	(Zhang, Yin, and Wang 2021)
(Large × White × Landrace) finishing pigs	\	Diet containing 0.02%, 0.04%, or 0.08% (w/w) chlorogenic acid	Daily feeding for 35 days	Improved antioxidant capacity and promoted myogenesis	Improving the amino acid composition of the longissimus dorsi (LD) and biceps femoris (BF) muscles, and increasing the mRNA abundance of Nrf‐2, GPX‐1, MyoD, MyoG, and oxidative muscle fibers (I and IIa) in the LD muscle	(Wang et al. 2021)
Duroc × Landrace × Yorkshire (DLY) pigs	\	Chlorogenic acid (25–100 mg/kg)	Fed three times daily for 100 days	Enhanced muscle antioxidant capacity and induced a shift in muscle fiber type toward slow‐twitch fibers	Decreasing the concentration of MDA in muscle tissue, while increasing the content of GSH‐Px in muscle and the levels of serum GSH‐Px, CAT, and T‐SOD	(Xie et al. 2023)
C57BL mice	Cardiotoxin (10 μM)	Diet containing 0.4% (w/w) resveratrol	Daily feeding for of 9 weeks	Improved TA muscle quality and stimulated regeneration by enhancing myogenesis and mitochondrial biogenesis	Upregulating the expression of proteins related to mitochondrial biogenesis (Tfam, Nrf1, Nrf2, Cpt1, Cycs) and enhancing expression of key myogenic regulators (YAP/p‐YAP, Pax7, Myf5, MyoG)	(Qin et al. 2025)
	\	Urolithin A (10 mg/kg)	Oral administration once daily for 12–16 consecutive weeks	Promoted angiogenesis in skeletal muscle	Enhancing ATP and NAD+ levels in skeletal muscle, activating the SIRT1‐PGC‐1α pathway to upregulate angiogenesis	(Ghosh et al. 2020)
	D‐galactose (100 mg/kg)	Highland barley tea polyphenols(4, 20 and 100 mg/kg)	Daily subcutaneous injections of d‐galactose and oral gavage of HBP for 9 weeks	Increased the mass of the soleus muscle and alleviated skeletal muscle atrophy	Reducing the serum levels of AGEs and enhancing the expression of Atrogin‐1 and SIRT3	(Nie et al. 2022)
	\	Apigenin (25, 50 or 100 mg/kg/d)	Oral gavage administration once daily for 9 months	Reduced oxidative stress, inhibited autophagy and apoptosis, alleviated skeletal muscle atrophy	Increasing ATP content, enhancing expression levels of PGC‐1α, TFAM, NRF‐1, and ATP5B, elevating activities of superoxide dismutase and glutathione peroxidase, improving total antioxidant capacity (T‐AOC)	(Wang, Yang, et al. 2020)
	Cisplatin (2.5 mg/kg)	Magnolol (1, 5 or 10 mg/kg)	Administered every 3 days for 30 days	Alleviated muscle atrophy	Mitigating cisplatin‐induced body weight and muscle loss, increasing CD163+ M2c macrophages and IGF‐1 expression	(Lee et al. 2020)
	Tail suspension	EGCG (5 mg/kg bw/d)	Orally administered once daily for 10 days	Inhibited muscle atrophy in the gastrocnemius	Suppressing the expression of MuRF1 mRNA, with eriodictyol enhancing the inhibitory effect of EGCG on disuse‐induced muscle atrophy	(Murata et al. 2020)
		EGCG and eriodictyol (5 mg/kg bw/d)		Prevented the loss of muscle mass in the gastrocnemius		
	HFD	Resveratrol (50, 100 or 200 mg/kg bw/d)	Fed daily for 4 consecutive weeks	Alleviated skeletal muscle atrophy, suppressed inflammation in skeletal muscle	Downregulating the expression of Bcl‐2 protein and MMP9, reduces the synthesis of ANP and BNP proteins, decreasing the expression of IL‐6	(Bai et al. 2020)
	Unilateral hind limb cast immobilization	Resveratrol (20 mg/kg bw/d)	Administer the medication daily for 14 consecutive days	Enhanced muscle regeneration, alleviated disuse atrophy	Increasing the number of satellite cells (muscle stem cells, quiescent cells, activated cells, and total satellite cells)	(Manas‐Garcia et al. 2020)
		Curcumin (1 mg/kg bw/d)				
Adult zebrafish ( *Danio rerio* )	Dexamethasone (0.01%)	Trifuhalol A (0.3%, 1%, 3%)	10 days	Maintained muscle structure, promoted myofiber recovery, and alleviated muscle atrophy	Enhancing muscle fiber cross‐sectional area and fiber density	(Yang et al. 2025)

**FIGURE 1:**
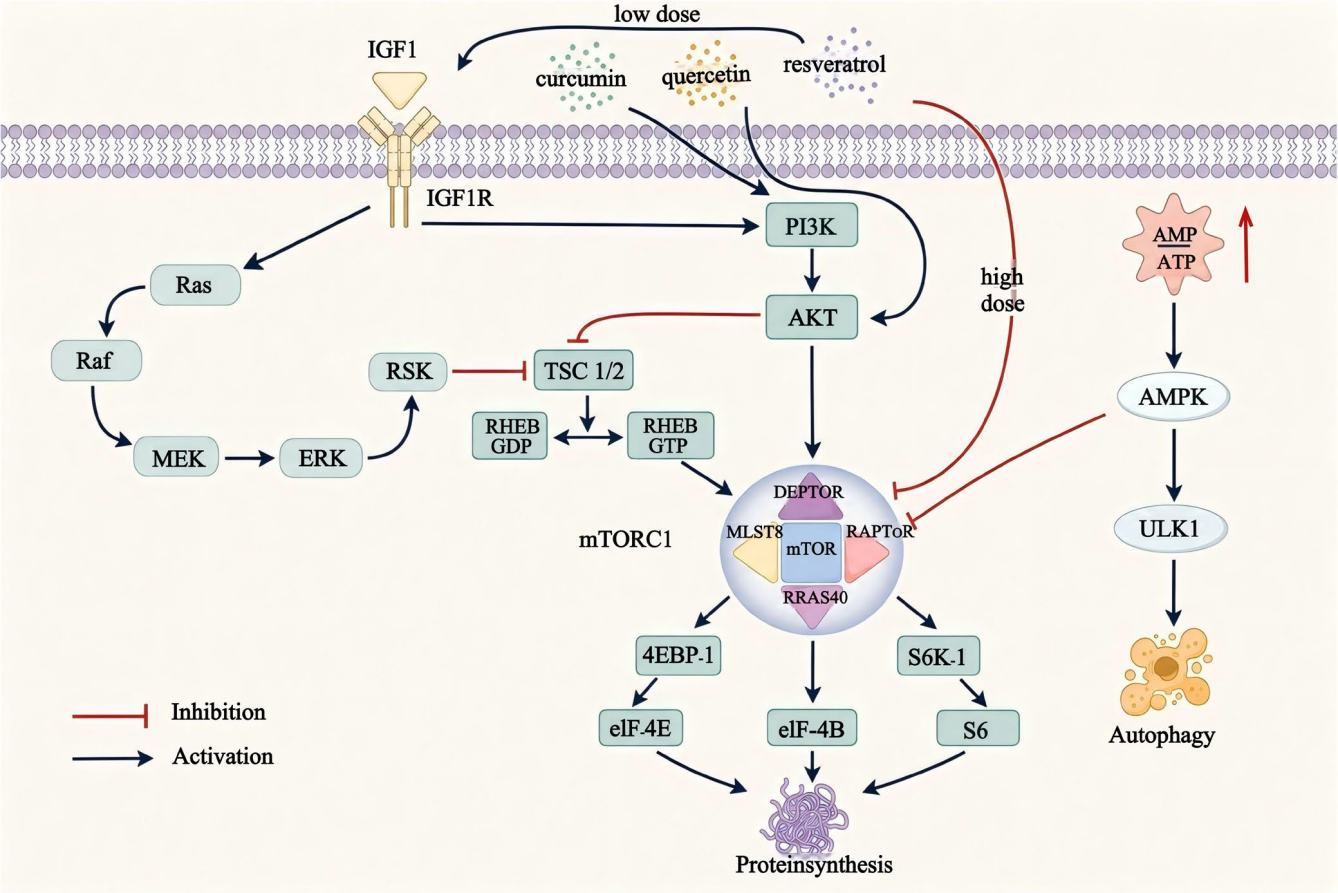
Mechanisms by which polyphenols regulate protein synthesis. Polyphenols, including curcumin, quercetin, and resveratrol, influence protein synthesis through the IGF‐1/PI3K/AKT/mTORC1 signaling pathway. Polyphenols, including curcumin, quercetin, and resveratrol, influence protein synthesis through the IGF‐1/PI3K/AKT/mTORC1 signaling pathway. At low levels, curcumin activates Ras, which triggers a cascade leading to ERK activation. Quercetin and resveratrol regulate this pathway by modulating PI3K/AKT signaling, thereby subsequently activating the mTORC1 complex. The mTORC1 pathway promotes protein synthesis by inhibiting 4EBP‐1 and activating S6K‐1, eIF4E, and eIF4B. Additionally, polyphenols influence autophagy through the AMPK/ULK1 pathway, with AMP levels affecting autophagic processes. 4EBP‐1, Eukaryotic translation initiation factor 4E‐binding protein 1; AKT, Protein kinase B; AMPK, AMP‐activated protein kinase; eIF4B, Eukaryotic translation initiation factor 4B; eIF4E, Eukaryotic translation initiation factor 4E; IGF‐1, Insulin‐like growth factor 1; mTORC1, Mechanistic target of rapamycin complex 1; PI3K, Phosphoinositide 3‐kinase; S6K‐1, Ribosomal protein S6 kinase 1; ULK1, Unc‐51‐like autophagy‐activating kinase 1.

### 2.2 Role of Polyphenols in the Regulation of Protein Degradation

The ubiquitin–proteasome system (UPS) and the autophagy–lysosomal system are the two principal pathways responsible for cellular protein degradation. The UPS predominantly removes short‐lived or abnormal proteins, whereas the autophagy–lysosomal system degrades protein aggregates, damaged organelles, and other larger cellular components (Dale et al. 2021; Li et al. 2022; Salucci and Falcieri 2020). Both pathways are required for normal protein quality control, but their excessive activation under catabolic conditions can contribute to muscle wasting.

Several polyphenols attenuate the activation of proteolytic markers in experimental models of muscle atrophy. Curcumin reduced the expression of the muscle‐specific E3 ubiquitin ligases MuRF1 and Atrogin‐1 in stress‐ or dexamethasone‐treated muscle cells (Lyu et al. 2023; Sani et al. 2021; Shao et al. 2024; Taengthong et al. 2022). Daidzein also alleviated cisplatin‐induced skeletal muscle atrophy, accompanied by modulation of the Glut4/AMPK/FoxO pathway and downregulation of MuRF1 and Atrogin‐1 (Zhang, Yin, and Wang 2021). These findings are consistent with suppression of excessive UPS activation. However, changes in ubiquitin‐ligase expression alone do not directly quantify proteasomal activity or whole‐muscle protein breakdown.

Polyphenol effects on autophagy are more context dependent. In denervated rat skeletal muscle, resveratrol reduced total proteolysis and the excessive activation of both proteasomal and lysosomal degradation, partly through PKA/CREB‐associated regulation (I S Júnior et al. 2023). In other experimental settings, resveratrol has increased autophagosome formation and promoted the clearance of damaged proteins and organelles (Gu and Han 2023). These observations are not necessarily contradictory: suppressing excessive lysosomal proteolysis during muscle wasting differs from restoring basal autophagic quality control. Moreover, increased autophagosome abundance does not by itself establish increased autophagic flux because it may also result from impaired lysosomal clearance.

Additional mechanisms may contribute to proteostasis. Cyanidin has been reported to limit Ca^2+^ leakage from the sarcoplasmic reticulum and attenuate calpain activation, potentially reducing the cleavage of sarcomeric and cytoskeletal proteins (Najjar and Feresin 2021). EGCG, quercetin, and curcumin may also influence heat‐shock proteins and molecular chaperones involved in protein folding and stress resistance (Guo et al. 2021; Storniolo et al. 2015; Yin et al. 2021). Their antioxidant effects may further reduce oxidative protein modification and the subsequent targeting of damaged proteins for degradation (Kim et al. 2021; Rudrapal et al. 2022). However, much of this evidence is based on stress markers or pathway‐associated proteins rather than direct measurements of protein turnover.

Overall, current evidence supports the ability of selected polyphenols to restrain excessive proteolysis while maintaining protein quality control under specific catabolic conditions. The direction of the response cannot be generalized across compounds, doses, tissues, and disease states. Future studies should combine pathway markers with measurements of proteasomal activity, autophagic flux, and tracer‐based protein synthesis and breakdown to determine whether the net outcome is genuine protein accretion. The principal degradation and quality‐control mechanisms are summarized in Figure 2.

**FIGURE 2:**
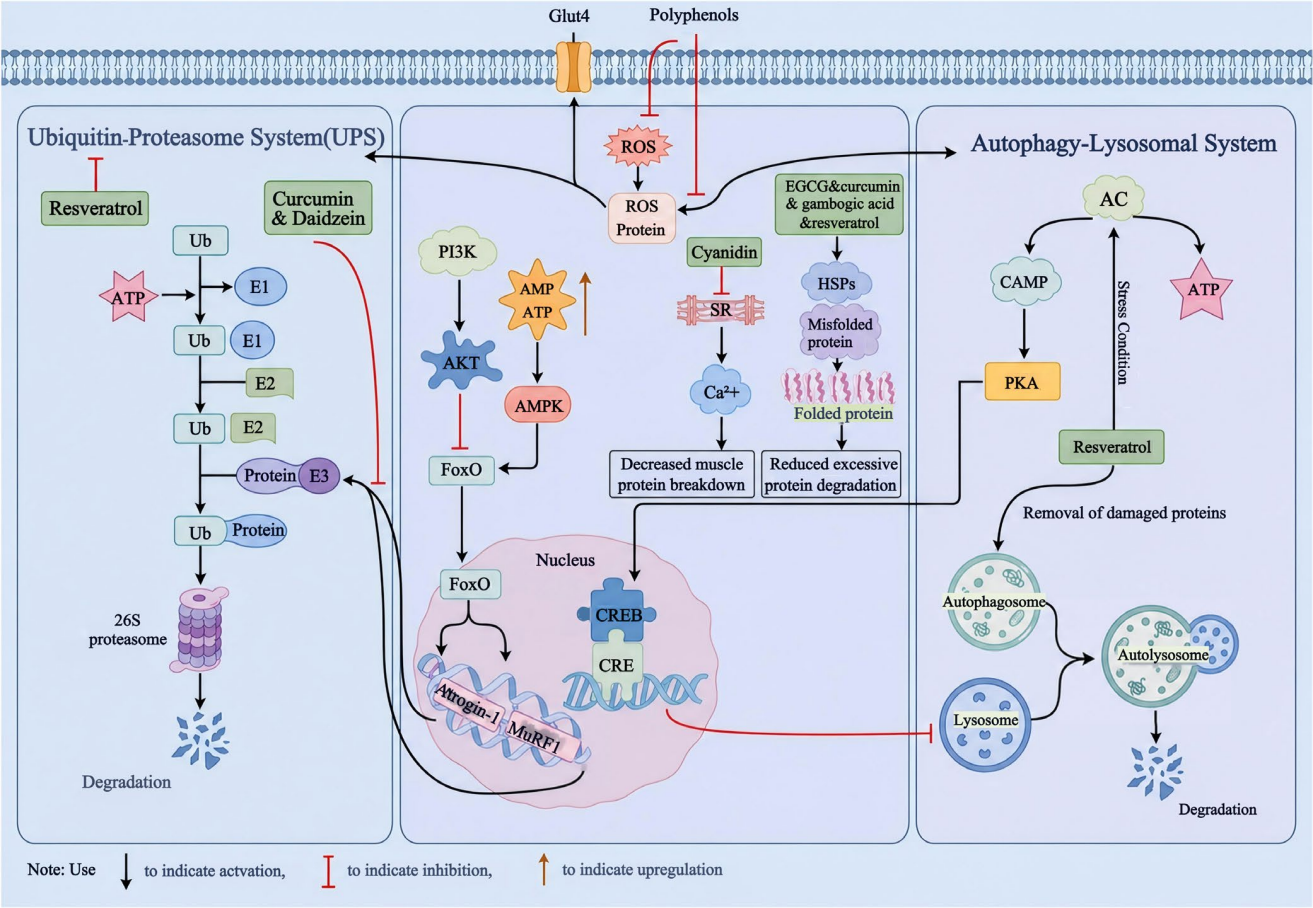
Mechanisms by which polyphenols regulate host protein degradation. Polyphenols, such as resveratrol, curcumin, daidzein, and cyanidin, regulate protein degradation through the ubiquitin‐proteasome system (UPS) and autophagy‐lysosomal system. In the UPS, polyphenols activate key enzymes (E1, E2, and E3) to tag proteins with ubiquitin (Ub), facilitating degradation via the 26S proteasome. Additionally, polyphenols influence reactive oxygen species (ROS) production, leading to sarcoplasmic reticulum (SR) modulation and protein breakdown. Polyphenols also regulate the PI3K/AKT/FoxO pathway, affecting muscle protein degradation. In the autophagy‐lysosomal system, polyphenols promote autophagosome formation and lysosomal degradation of damaged proteins. AC, Adenylyl cyclase; AKT, Protein kinase B; AMP, Adenosine monophosphate; AMPK, AMP‐activated protein kinase; ATP, Adenosine triphosphate; cAMP, Cyclic adenosine monophosphate; CRE, CAMP response element; CREB, CAMP response element‐binding protein; FoxO, Forkhead box O transcription factors; Glut4, Glucose transporter type 4; HSPs, Heat shock proteins; MURF1, Muscle‐specific ring finger protein 1; PI3K, Phosphoinositide 3‐kinase; PKA, Protein kinase A; ROS, Reactive oxygen species; SR, Sarcoplasmic reticulum.

## 3 Indirect Metabolic Influences on Protein Deposition

Beyond their direct effects on protein synthesis and degradation, polyphenols may also shape net protein deposition through broader metabolic adjustments, including amino acid availability, nutrient partitioning, and glucose utilization. The PI3K/Akt–mTORC1 and AMPK signaling pathways underlying these direct regulatory effects have been detailed in Section 2 and are not repeated here. Building on this framework, Figure 3 summarizes the main indirect metabolic routes through which polyphenols may act. In the following sections, we evaluate whether these reported metabolic shifts are consistently associated with changes in muscle composition, protein accretion, or growth performance. Notably, much of the existing evidence is based on transporter expression profiles, tissue composition indices, serum biomarkers, or growth outcomes, rather than direct quantification of metabolic fluxes or protein turnover rates.

**FIGURE 3:**
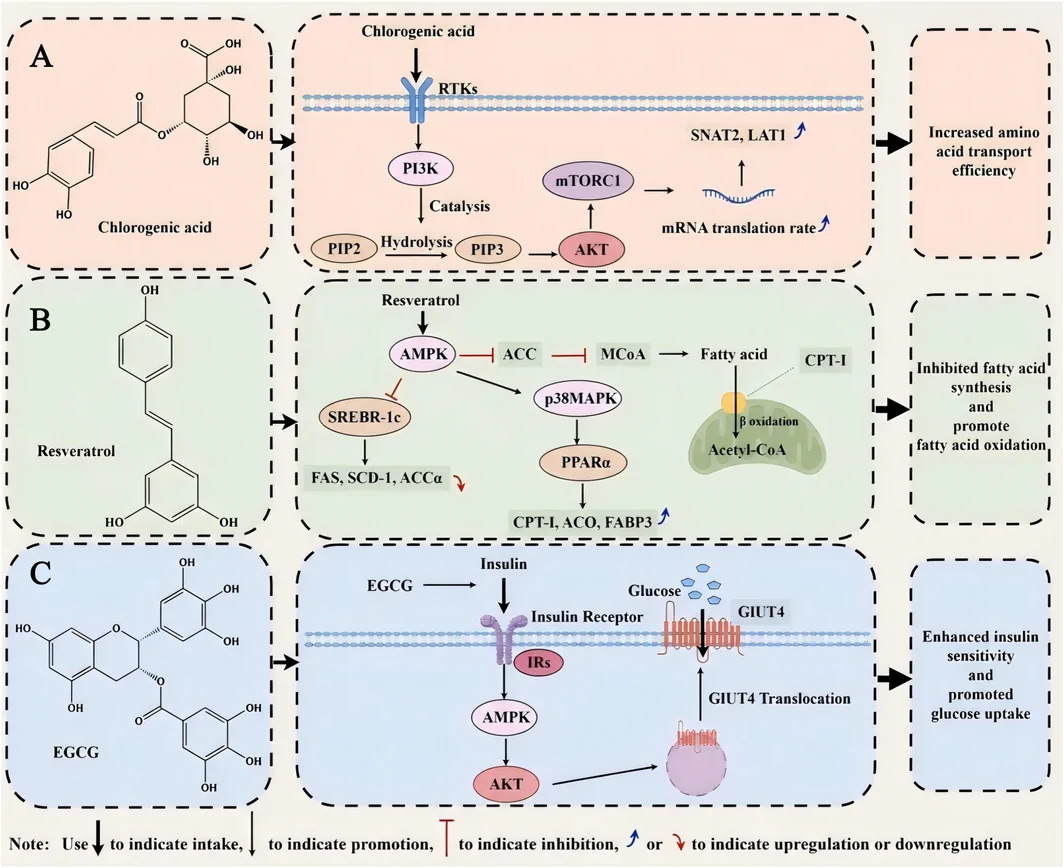
Mechanisms by which polyphenolic compounds regulate metabolic pathways: (A) Regulation of amino acid transport to enhance protein synthesis efficiency: Activation of the PI3K/AKT pathway increases amino acid transport efficiency. (B) Regulation of lipid metabolism: Through the AMPK signaling pathway, polyphenols inhibit lipogenesis while enhancing fatty acid β‐oxidation. (C) Improvement of glucose metabolism: Activation of the PI3K/AKT pathway enhances GLUT4 translocation, accelerating glucose uptake and utilization. ACC, Acetyl‐CoA carboxylase; CPT‐1, Carnitine palmitoyltransferase 1; FABP3, Fatty acid‐binding protein 3; FAS, Fatty acid synthase; GLUT4, Glucose transporter type 4; LAT1, L‐type amino acid transporter 1; MCoA, Malonyl‐CoA; RTKs, Receptor tyrosine kinase; SCD‐1, Stearoyl‐CoA Desaturase‐1; SNAT2, Sodium‐coupled neutral amino acid transporter 2; SREBP‐1c, Sterol‐regulatory element binding protein‐1c.

### 3.1 Polyphenol Interactions With Amino Acid Transporters

As the building blocks of proteins, amino acids are indispensable substrates for protein synthesis. The availability of essential amino acids, particularly the branched‐chain amino acids leucine, isoleucine, and valine, is especially important for stimulating skeletal muscle protein synthesis (Gorissen and Phillips 2019). Dietary supplementation with apple polyphenols at 400 or 800 mg/kg of diet for 7 weeks altered muscle amino acid composition in finishing pigs. At 800 mg/kg, apple polyphenols increased crude protein content and the proportions of essential, flavor, and total amino acids in the longissimus dorsi muscle, together with increased mRNA expression of the amino acid transporters SLC7A1, SLC7A2, SLC7A7, and SLC1A2 (Xu, Chen, et al. 2019). These findings are consistent with altered amino acid transport capacity and muscle composition. Nevertheless, transporter mRNA abundance does not directly demonstrate increased transporter activity, amino acid flux, or incorporation of amino acids into newly synthesized muscle protein.

Chlorogenic acid supplementation similarly increased total and flavor amino acid contents and altered MyHC IIa and MyHC IIX expression in the longissimus thoracis muscle of finishing pigs (Xie et al. 2023). These outcomes indicate changes in muscle composition and fiber‐type‐related gene expression, but they do not establish whether chlorogenic acid increased net protein deposition. In grass carp, dietary curcumin or ferulic acid increased the expression of intestinal amino acid and peptide transporters and was accompanied by improved growth performance (Li et al. 2020; Shao et al. 2024). Again, these studies primarily assessed transporter transcripts and growth rather than intestinal amino acid absorption rates or muscle protein synthesis.

Evidence in humans remains particularly limited. Studies are needed that combine controlled polyphenol interventions with measurements of postprandial amino acid availability, muscle amino acid uptake, and tracer‐derived protein synthesis. Until such evidence is available, regulation of amino acid transport should be regarded as a plausible but incompletely demonstrated contributor to polyphenol‐associated protein deposition.

### 3.2 Polyphenol Interactions With Lipid Metabolism

Lipid metabolism is closely linked to body composition because it determines how nutrients are partitioned between fat storage, energy production, and lean‐tissue accretion. Polyphenols have been reported to modulate lipogenesis, lipolysis, and fatty acid oxidation, potentially reducing excessive lipid deposition and altering the metabolic environment for protein accretion (Niu et al. 2024; Zou et al. 2024).

In the finishing‐pig study described above, 800 mg/kg dietary apple polyphenols reduced backfat thickness and the abdominal adipose tissue index while increasing muscle crude protein content (Xu, Li, et al. 2019). Because the lipid and protein outcomes were measured concurrently, the study cannot determine whether reduced adiposity directly caused greater muscle protein deposition. Dose dependence is also important. In juvenile black carp, a moderate dose of tea polyphenols (50 mg/kg) improved growth performance and crude protein content while reducing crude lipid content, whereas the highest tested dose reduced growth and crude protein content (Zhong et al. 2020). This non‐linear response argues against treating higher polyphenol exposure as uniformly beneficial and highlights the need to establish species‐ and compound‐specific dose ranges.

Limited human evidence provides similarly indirect associations. In an open‐label, single‐arm study involving 46 older adults, consumption of a fermented black soybean product containing polyphenols for 10 weeks was associated with attenuation of skeletal muscle loss and reduced serum low‐density lipoprotein concentrations (Chang et al. 2023). However, the absence of a concurrent control group prevents separation of the intervention effect from temporal, dietary, or behavioral changes. Moreover, the simultaneous improvement in muscle and lipid outcomes does not demonstrate that altered lipid metabolism mediated muscle preservation. Controlled trials with direct body‐composition and protein‐turnover measurements are therefore required.

### 3.3 Glucose Utilization and Insulin Responsiveness

Carbohydrates provide an important energy source for protein turnover and lean‐tissue growth, while glucose homeostasis determines how efficiently this substrate is delivered to and utilized by peripheral tissues. Polyphenols may influence carbohydrate metabolism by improving insulin sensitivity, promoting glycogen synthesis, and modulating intestinal glucose absorption (Williamson and Sheedy 2020). Certain phenolic compounds can also inhibit α‐amylase and α‐glucosidase, thereby slowing starch digestion and attenuating postprandial glycaemic excursions (Aleixandre et al. 2022; Zhang et al. 2022). Together, these actions may alter glucose availability and utilization in skeletal muscle and thus modify the energetic conditions supporting protein turnover and deposition.

At the cellular level, physiologically relevant concentrations of resveratrol and quercetin have been shown to modulate glucose metabolism in primary human myotubes (Eseberri et al. 2021). Theaflavin promoted mitochondrial abundance and glucose absorption in C2C12 myotubes (Qu et al. 2021), whereas EGCG enhanced glucose uptake and GLUT4 translocation in L6 skeletal muscle cells (Xu, Chen, et al. 2019).

Evidence from livestock and aquaculture further suggests that polyphenols may affect carbohydrate digestion and utilization. In finishing pigs, dietary chlorogenic acid increased intestinal expression of SGLT1 and GLUT2 and altered serum albumin, IGF‐1, and urea nitrogen concentrations (Chen et al. 2018). In grass carp, supplementation with 500 mg/kg tea polyphenols increased the expression of genes and activities of enzymes involved in carbohydrate degradation, accompanied by improved carbohydrate utilization and growth performance (Yue et al. 2021). A diet enriched with polyphenolic extract from olive‐mill wastewater similarly improved carbohydrate efficiency and growth indices in crayfish (Parrillo et al. 2017).

Taken together, the available evidence suggests that polyphenols can influence amino acid availability, lipid partitioning, and glucose utilization in ways that may support protein deposition. However, the evidence currently demonstrates mainly the co‐occurrence of metabolic and growth‐related changes rather than causal mediation. In particular, enhanced glucose uptake in cultured muscle cells, altered transporter expression, serum biomarkers, digestive‐enzyme activities, and growth performance do not independently establish increased muscle protein synthesis. Future studies should therefore combine measurements of nutrient flux and insulin responsiveness with body‐composition analysis, energy‐matched or pair‐fed controls, and tracer‐based measurements of protein synthesis and breakdown to determine whether these metabolic effects produce genuine net protein accretion.

## 4 Common Models for Studying Polyphenols in Protein Metabolism, Deposition, and Muscle Health

The effects of polyphenols on skeletal muscle protein metabolism and deposition have been investigated using a variety of research platforms, each offering unique insights into the underlying mechanisms. These platforms include in vitro cell models, animal experiments, and human studies, each with distinct advantages and limitations. This section provides a comprehensive overview of these models, detailing their key characteristics, commonly used induction agents, polyphenol types, dosages, study outcomes, and the primary mechanisms involved. Tables 1, 2, 3 summarize the different study types on the regulation of protein metabolism and skeletal muscle protein deposition, highlighting the mechanisms that underpin these effects.

**TABLE 3:** Human studies on the effects of dietary polyphenols on protein regulation and muscle health.

Subjects	Experimental setting	Polyphenol and dosage	Duration	Effect	Key mechanism	References
Healthy, recreationally active adults; PPB, *n* = 15; placebo, *n* = 14	Resistance training	Protein‐polyphenol beverages (containing 480 mg of tart cherry extract)	10–11 weeks	Accelerated the recovery from muscle damage, enhanced daily myofibrillar protein synthesis	Increasing the Type II fCSA of the vastus lateralis, enhancing the myofibrillar protein synthesis rate	(Pavis et al. 2022)
Elderly participants (total of 46, age: 76 ± 5.9; 11 males, calf circumference 33.4 ± 0.4 cm; 35 females, calf circumference 31.7 ± 1.0 cm); BMI < 18.5: 7 participants, 18.5–22.9: 12 participants, 23.0–24.9: 10 participants, 25.0: 17 participants	Open‐label, single‐arm trial with a pre–post study design	Fermented black soybean koji product (2 packets/day, each containing 44.9 mg of polyphenols)	10 weeks	Increased the appendicular skeletal muscle mass index, mitigated age‐related skeletal muscle loss	Reducing low‐density lipoprotein levels, enhancing the activity of antioxidant enzymes, altered the beta diversity of the gut microbiota, inducing SCFAs	(Chang et al. 2023)

### 4.1 In Vitro Cellular Studies

In vitro cell models are widely used to study the direct effects of polyphenols on skeletal muscle cell metabolism, particularly in the regulation of cellular signaling pathways and the dynamic balance between protein synthesis and degradation. These models allow researchers to efficiently assess the effects of polyphenols under tightly controlled experimental conditions. Common in vitro cell lines used to study skeletal muscle protein metabolism and deposition include the mouse C2C12 and rat L6 muscle cell lines, the immortalized human LHCN‐M2 cell line, and primary human skeletal muscle cells (HSkM) (Bałaban et al. 2023; Hour et al. 2022; Park and Han 2022; Turner et al. 2023). Among these, the C2C12 cell line is particularly popular due to its extensive use in research on muscle differentiation, repair, and metabolic regulation (Hour et al. 2022; Powrózek et al. 2022; Sani et al. 2021).

C2C12 cells are widely used not only because they are well established, but also because they offer several practical advantages. These immortalized myoblasts can be readily expanded under standard growth conditions and differentiated into multinucleated myotubes within approximately 4–7 days after switching to low‐serum medium. This relatively short and reproducible workflow is compatible with multiwell‐plate experiments, allowing concentration–response and time‐course screening with modest cell and reagent requirements. C2C12 cells are also amenable to gene knockdown, overexpression, immunofluorescence, and biochemical assays of protein synthesis and degradation (Cocchiararo et al. 2022). Model selection should nevertheless depend on the experimental endpoint. C2C12 cells are particularly useful for studying myogenic differentiation, myotube atrophy, and contractile‐protein regulation, whereas L6 cells are frequently selected for insulin responsiveness and glucose‐uptake studies. Primary human myotubes provide greater species relevance but require donor tissue, have limited expansion capacity, and show greater donor‐to‐donor and differentiation variability. Generally, in vitro cell assays offer advantages such as short experimental timelines, ease of manipulation, high reproducibility, precise control over conditions, and no ethical concerns related to animal research. Commonly used induction agents in these cell assays include oxidative stressors (e.g., H_2_O_2_) (Ceci et al. 2022) and inflammatory cytokines (e.g., TNF‐α) (Powrózek et al. 2022), which are used to mimic pathological conditions in skeletal muscle, allowing researchers to assess the antioxidant and anti‐inflammatory effects of polyphenols in muscle cell protection at the cellular level (Zhang et al. 2023). Table 1 summarizes in vitro studies on the positive effects of dietary polyphenols on protein regulation and muscle health (Studies before 2020 are provided in Table S1).

Although in vitro models cannot directly infer how polyphenols influence whole‐body protein deposition, they provide valuable mechanistic insights into the cell‐autonomous regulation of muscle protein synthesis and degradation. Therefore, in vitro cell assays provide valuable insights into how protein metabolism may be affected when muscle cells are influenced by polyphenols. However, in vitro models may not fully replicate the complex physiological conditions in vivo, as cellular responses in simple assays can differ from those involving intercellular interactions, tissue architecture, and integrated metabolic effects. Recent advancements in in vitro models, such as co‐culture systems (David et al. 2023; Jafarkhani et al. 2023), 3D cell cultures (Dessauge et al. 2021), and organoids (Jalal et al. 2021), are gaining traction as innovative research platforms. By better reproducing tissue architecture and cellular interactions, these models may improve the investigation of polyphenol effects on skeletal muscle protein metabolism under complex physiological or pathological conditions. From an operational perspective, a tiered validation strategy may provide a more efficient model design. C2C12 cells can first be used for concentration–response screening and preliminary target identification, after which active treatments should be validated in primary human myotubes and, where relevant, in co‐culture or 3D muscle constructs. Parent polyphenols should also be compared with their circulating or microbial metabolites at concentrations consistent with measured in vivo exposure. In addition to pathway‐associated proteins, studies should incorporate functional measurements such as amino acid incorporation or puromycin‐based protein‐synthesis assays, proteasomal activity, autophagic flux, myotube diameter, and contractile function. This stepwise approach would retain the experimental efficiency of C2C12 cells while progressively improving physiological relevance. Nevertheless, greater structural complexity does not overcome limitations related to the use of unmetabolized parent compounds or concentrations that may not be achieved in vivo. Therefore, in vitro models should primarily be regarded as platforms for mechanistic investigation, and their findings require validation in animal or human studies.

### 4.2 Laboratory Animal Models

Animal models are commonly used to investigate the effects of polyphenols on skeletal muscle protein metabolism and deposition, offering a more physiologically relevant environment than in vitro models. Mice, particularly the C57BL/6 strain (Kim et al. 2025; Matsuzaki et al. 2025; Nikawa et al. 2021), are commonly employed in studies of muscle atrophy and injury, including drug‐induced damage, exercise‐induced injury, and inflammation‐related atrophy. Their short experimental timelines, well‐defined genetic backgrounds, and suitability for mechanistic studies make them ideal for exploring the impact of polyphenols on muscle function. Rats are also widely used due to their larger size, which facilitates intragastric administration, intravenous injection, tissue sampling, and long‐term observation (Aslan et al. 2020; Liu et al. 2021; Ommati et al. 2020; Sahin et al. 2024). Administered doses should not be compared directly across animal species or translated to humans solely on a mg/kg body‐weight basis. Body‐surface‐area scaling may provide a preliminary human‐equivalent dose for mammalian studies, but it does not account for species differences in gastrointestinal physiology, gut microbiota, polyphenol absorption, conjugative metabolism, clearance, or tissue distribution. Dietary exposures expressed as mg/kg of feed also require consideration of feed intake, body weight, and intervention duration before comparison with gavage doses or human intake. Cross‐species extrapolation should therefore prioritize measured plasma or tissue concentrations and species‐specific physiologically based kinetic models. The translational relevance of an animal model also depends on how closely its physiology resembles that of humans. In this context, pigs have gained attention as a valuable resource for biomedical research due to their greater physiological similarities to humans compared to rodents, particularly in drug metabolism and disease progression (Niu and Yin 2024; Wang et al. 2024). These characteristics provide a more physiologically relevant setting for investigating how polyphenols influence muscle protein metabolism and deposition (Wang et al. 2025). However, the high cost, greater housing requirements, and longer experimental timelines associated with pig models present challenges for their widespread application in basic research. Table 2 illustrates animal models for studying the effects of dietary polyphenols on protein regulation and muscle health (Studies before 2020 are provided in Table S2).

### 4.3 Human Studies

Human studies provide the most direct evidence for evaluating the effects of dietary polyphenols on skeletal muscle protein metabolism and muscle health (Paoli et al. 2024; Pavis et al. 2022). The most common approach in human studies is dietary intervention, where participants are provided with polyphenol‐rich foods (e.g., green tea, wine, blueberries) or polyphenol supplements to evaluate their impact on protein utilization, muscle health, strength, and endurance (Paoli et al. 2024). Dietary intervention studies and randomized controlled trials (RCTs) offer the advantage of assessing the real‐world effects of polyphenols as part of daily nutrition, providing strong evidence for their role in human health. These studies typically follow well‐defined guidelines and protocols, including participant numbers, study duration, dietary restrictions, and monitoring for potential confounding factors (Lichtenstein et al. 2021; Maki et al. 2021). In addition, researchers monitor for potential confounding factors, such as baseline health conditions (e.g., obesity, diabetes), physical activity levels, and lifestyle factors (e.g., smoking, alcohol consumption), which could influence study outcomes (Thomas and Hodges 2020). Recruitment, retention, adherence, and interindividual variability remain important challenges, particularly in older adults and patients with muscle‐related disorders (Chang et al. 2023; Favari et al. 2024). In addition to dietary interventions and RCTs, epidemiological studies, such as large‐scale cohort studies, can provide valuable insights into the long‐term correlation between polyphenol intake and muscle health outcomes (Marino et al. 2020; O'Leary et al. 2025). Table 3 presents human studies on the effects of dietary polyphenols on protein regulation and muscle health.

Despite their direct relevance, human studies are constrained by adherence, uncertainty in actual intake and internal exposure, heterogeneous intervention composition, background diet, and interindividual differences in polyphenol metabolism (Condezo‐Hoyos et al. 2021; Favari et al. 2024). Long follow‐up periods may also make these studies resource‐intensive and costly. Consequently, a null result may reflect inadequate exposure rather than biological inactivity, whereas a positive response to a polyphenol‐rich food or multicomponent supplement cannot necessarily be attributed to a specific polyphenol. Existing pharmacokinetic and clinical data could be integrated with New Approach Methodologies (NAMs) to make the translation from cell‐based findings to human trials more quantitative (Liu et al. 2023). One practical approach is quantitative in vitro‐to‐in vivo extrapolation (QIVIVE), in which concentration–response data from muscle‐cell assays are linked to a physiologically based kinetic (PBK) model (Liu, Meng, et al. 2022). Rather than directly converting the nominal concentration added to the culture medium into an oral dose, investigators should first determine or estimate the unbound concentration and obtain compound‐specific inputs for intestinal absorption, intrinsic hepatic clearance, plasma protein binding, and tissue‐to‐plasma distribution. Quantitative structure–activity relationship (QSAR) models can help prioritize potentially active parent compounds and metabolites, while in silico calculators can estimate missing physicochemical or kinetic parameters (Singh et al. 2026). For example, the web‐based toolbox developed by Punt et al. (2021) integrates generic human and rat PBK models with calculators for plasma protein binding and tissue‐to‐plasma partitioning. Through reverse dosimetry, it can estimate the oral‐equivalent dose required to reproduce an internal concentration associated with an in vitro effect.

Although this toolbox was developed primarily for nonanimal chemical assessment rather than nutritional efficacy, its QIVIVE framework could be adapted to identify physiologically plausible polyphenol exposures before initiating human trials. For polyphenols, the analysis should include not only the administered parent compounds but also their major circulating conjugates and gut microbial metabolites, because these forms may dominate systemic exposure (Liu, van Mil, et al. 2022; Liu et al. 2020). Predicted oral‐equivalent doses should then be compared with measured plasma concentrations, concentration–time profiles, and urinary or plasma metabolite data from previous human studies. If the internal concentration required to reproduce an in vitro effect cannot be achieved through realistic dietary or supplemental intake, that finding should not be advanced directly to a clinical efficacy trial. Conversely, physiologically plausible predictions could guide dose selection, intervention timing relative to protein intake or exercise, and blood‐ or muscle‐sampling windows. Sensitivity and uncertainty analyses should also be used to identify whether predictions depend strongly on poorly characterized absorption, clearance, protein‐binding, or tissue‐distribution parameters that require experimental measurement.

The subsequent clinical design should reflect the specific research question. Acute crossover studies can assess postprandial muscle protein synthesis using stable‐isotope‐labeled amino acids, whereas longer‐term parallel trials can use deuterated water to measure integrated muscle protein synthesis over several days or weeks (Holwerda et al. 2024). When polyphenols are administered together with protein supplements or exercise, factorial designs should be used to distinguish their independent and interactive effects. These measurements should be combined with prespecified outcomes such as muscle mass assessed by dual‐energy X‐ray absorptiometry or magnetic resonance imaging, muscle strength, and physical function. Such an integrated strategy would provide stronger translational evidence than trials based only on administered dose, circulating biomarkers, or changes in body weight.

## 5 Applications of Polyphenols in Animal Production and Human Health

As discussed above, polyphenols play a crucial role in regulating protein metabolism, promoting muscle health, and improving meat quality. This section reviews the diverse applications of polyphenols in enhancing animal production systems and advancing human health, emphasizing their potential as natural bioactive compounds to optimize protein deposition, improve livestock performance, enhance meat quality, and contribute to disease prevention and overall well‐being in humans (Figure 4).

**FIGURE 4:**
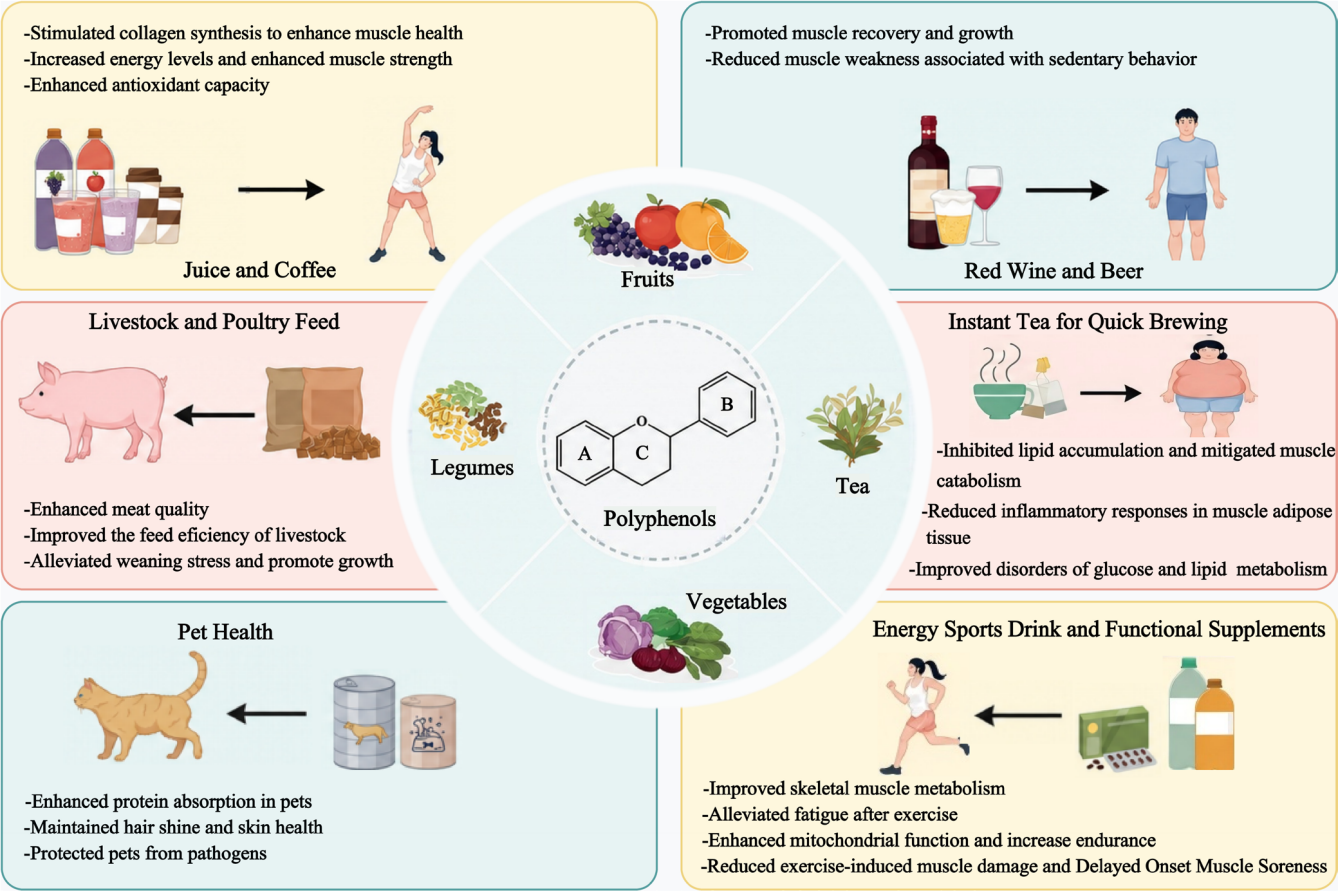
Applications of polyphenols in livestock farming and human health. Polyphenols have various beneficial effects in both animal and human health, as illustrated across multiple sectors: Juice and Coffee: Enhance antioxidant capacity and energy levels, supporting improved muscle strength and overall health. Livestock and Poultry Feed: Improve meat quality, feed efficiency, alleviate weaning stress, and promote growth. Pet Health: Enhance protein absorption, maintain fur shine and skin health, and protect pets from pathogens. Fruits: Promote muscle recovery, growth, and reduce muscle weakness associated with sedentary behavior. Legumes: Stimulate collagen synthesis, enhancing muscle health. Tea: Alleviate fatigue after exercise, improve skeletal muscle metabolism, and enhance mitochondrial function. Vegetables: Support muscle metabolism, reduce exercise‐induced damage, and alleviate delayed onset muscle soreness. Red Wine and Beer: Promote muscle recovery and growth, while reducing muscle catabolism. Instant Tea for Quick Brewing: Inhibit lipid accumulation and mitigate muscle catabolism, improving metabolic health. Energy Sports Drinks and Functional Supplements: Support endurance, reduce inflammatory responses, and improve glucose and lipid metabolism.

### 5.1 Applications of Polyphenols in Animal Husbandry

Polyphenols have been investigated as potential feed additives for improving nutrient utilization, stress resilience, production performance, and animal‐product quality. Specifically, grape extract polyphenols have been shown to significantly enhance amino acid utilization in broiler chickens, mitigating the reduced digestibility caused by coccidiosis (Chalvon‐Demersay et al. 2021). In finishing pigs, dietary polyphenols have been associated with increased favor amino acids, essential amino acids, and total amino acids in the LD muscle (Wang et al. 2021; Wang, Li, et al. 2022). They also promote the deposition of muscle crude protein (Xu et al. 2022), intramuscular fat and inosinic acid (Wang, Wang, et al. 2020), contributing to improved meat quality and feed conversion efficiency (Mei et al. 2024). Polyphenol‐rich fruit extracts and agro‐industrial by‐products, have also shown notable benefits in ruminants like cattle, goat and sheep (Bešlo et al. 2022; Formato et al. 2022). Adding dietary polyphenol supplements to cow and cattle diets improves protein metabolism, resulting in increased milk fat and protein content, thereby enhancing the nutritional value of dairy products (Herremans et al. 2020; Nair et al. 2025; Olagaray et al. 2019). Furthermore, the antioxidant and anti‐inflammatory properties of polyphenols help reduce the incidence of metabolic diseases, such as fatty liver, and mitigate the negative effects of environmental stress on animals (Ma et al. 2021; Yan et al. 2020). For instance, under heat stress conditions, polyphenol‐enriched diets, such as those containing puerarin, have been shown to effectively reduce serum cortisol levels, thereby mitigating stress responses (Peng et al. 2021).

Nevertheless, the effects of polyphenols vary with their source and dose, as well as with animal species, physiological status, and production conditions. Improvements in growth performance or meat quality do not necessarily indicate increased protein deposition. Targeted supplementation under defined production conditions is therefore more realistic than broad application. Development should begin with chemically characterized ingredients and marker compounds, followed by species‐specific dose–response studies incorporating direct measurements of protein deposition and evaluations of processing and storage stability, feed‐matrix interactions, actual intake, and batch consistency under commercial conditions. Although agro‐industrial by‐products may reduce costs and support circular production, these benefits must be balanced against compositional variability, quality‐control requirements, palatability, contaminants, and antinutritional constituents. Moreover, natural origin alone does not establish safety or regulatory acceptability; authorization may require evidence of efficacy and safety for animals, consumers, users, and the environment (European Parliament and Council, EFSA FEEDAP Panel (EFSA Panel on Additives and Products or Substances used in Animal Feed) (2024); European Union 2003). Commercial feasibility should ultimately be assessed by balancing additive costs against returns from feed efficiency, animal health, carcass or product quality, while also considering consumer acceptance.

### 5.2 Applications of Polyphenols in Human Nutrition

In the field of human health promotion, polyphenols have garnered significant attention for their role in improving metabolic health and supporting protein synthesis (Figure 4). Particularly in addressing age‐related sarcopenia (Medoro et al. 2024), chronic diseases (Shanmugam 2025), and malnutrition, polyphenols regulate key metabolic pathways, such as mTOR and AMPK, to optimize the balance between protein synthesis and degradation (Nikawa et al. 2021), providing a scientific foundation for exercise‐induced fatigue (Nikawa et al. 2021). For instance, Liu et al. demonstrated that *Lonicera caerulea* berries can alleviate long‐term exercise‐induced muscle damage by promoting mitochondrial biogenesis in skeletal muscle via modulation of the AMPK–PGC1‐α–NRF1–TFAM signaling axis, and by activating the miRNA‐133a/IGF‐1/PI3K/Akt/mTOR signaling pathway to enhance skeletal muscle myocyte proliferation (Liu, Meng, et al. 2022). The incorporation of polyphenols as active ingredients in functional foods is gaining popularity as a promising approach to deliver various health benefits (Aloo et al. 2023; Ma and Chen 2020), such as promoting protein synthesis, enhancing metabolic health, and supporting muscle maintenance and recovery (Chang et al. 2023). Polyphenol‐rich diets not only support muscle health but may also offer benefits in weight management (Boccellino and D'Angelo 2020), glucose regulation (Sun and Miao 2020), and cardiovascular health (Li et al. 2023; Mehmood et al. 2020), making them an attractive option for a broad population. However, despite extensive research on polyphenols' effects on body composition and insulin sensitivity (Wu et al. 2022), their specific application in enhancing protein deposition across diverse populations remains inconclusive.

Future studies should focus on exploring the protein metabolic effects of polyphenols in special populations (Quesada‐Vázquez et al. 2024), such as elderly individuals and athletes, to determine optimal dosages and synergistic effects with traditional protein supplements (Chang et al. 2023). Trials should directly quantify muscle protein synthesis and breakdown, preferably using stable‐isotope tracer methods, and determine whether these changes translate into improved muscle mass, strength, mobility, or exercise recovery. The formulations should also be evaluated for stability during processing and storage, sensory acceptability, long‐term adherence, production cost, and affordability. Safety, labelling, and the evidence required to support muscle‐health claims should be considered under the relevant regulatory framework. Such evidence is needed before targeted nutritional recommendations can be made.

## 6 Conclusions and Future Perspectives

This review synthesizes current evidence on how dietary polyphenols may influence protein synthesis, degradation, and net deposition in skeletal muscle. Special attention is given to how polyphenols modulate key signaling pathways such as AMPK, mTOR, and PI3K/Akt to regulate protein synthesis and degradation in skeletal muscle. Their antioxidant and anti‐inflammatory activities may also help preserve muscle under oxidative, inflammatory, or metabolic stress. In livestock, polyphenols may affect growth performance, muscle composition, and post‐mortem meat quality.

Despite substantial evidence supporting the positive effects of polyphenols on protein metabolism, protein deposition, and muscle health, several challenges remain. Variations in the source, dose, and duration of polyphenol interventions limit the comparability of results, particularly between in vitro, animal, and human studies, where differences in polyphenol intake and experimental design complicate direct comparisons. Additionally, issues such as individual variability, sample size limitations, and the lack of standardized polyphenol dosing persist in this field. In livestock studies, further work is needed to determine the optimal dose and application method of polyphenol additives, considering economic and efficacy factors to maximize the overall value of animal production.

Future research should focus on addressing these challenges and optimizing study designs. For livestock studies, particular attention should be paid to defining the optimal dose and application method of polyphenol additives based on a balance of economic and production outcomes. Standardizing polyphenol doses, intervention durations, and sources will improve comparability across studies and advance the field toward greater consistency. Moreover, the adoption of novel experimental models, such as organoid models and 3D cell culture systems, will provide more physiologically relevant platforms to investigate polyphenol mechanisms in protein regulation, metabolic interactions, and deposition. The advancement of personalized medicine will also help uncover varying responses to polyphenols among different populations (e.g., elderly individuals, athletes, and patients), promoting more precise and targeted interventions.

## Author Contributions

**Chen Liu:** writing – review and editing, writing – original draft, conceptualization. **Mengyi Ren:** data curation, formal analysis, software. **Xin Lai:** methodology. **Ke Gu:** data curation. **Liqiang Tan:** supervision, methodology. **Aimin Wu:** resources, supervision. **Daiwen Chen:** writing – review and editing, funding acquisition, supervision.

## Funding

This work was supported by National Key Project of the National Natural Science Foundation of China: “Molecular Mechanisms of Enhancing Protein Deposition in Pigs through Coordinated Nutrient Transport in the Diet” [Grant No. 2024NSFSC0297] and Research and Innovation in the Development of Low‐Fluoride High‐Quality Tibetan Tea Products [Grant No. LSKJ202534].

## Disclosure

*Declaration of AI and AI‐assisted technologies in the writing process*: During the preparation of this work, the author(s) used ChatGPT in order to improve the language and readability of the manuscript. After using this tool/service, the author(s) reviewed and edited the content as needed and take full responsibility for the content of the published article.

## Ethics Statement

Authors declare that the current study did not use any experimental animals and there are no human subjects involved in it. Therefore, the current study has no ethical issue.

## Conflicts of Interest

The authors declare no conflicts of interest.

## Supporting information


**Table S1:** In vitro cell models for studying the positive effects of dietary polyphenols on protein regulation and potential muscle health.
**Table S2:** Animal models for studying the effects of dietary polyphenols on protein regulation and muscle health.

## Data Availability

No new data were generated or analyzed in support of this review article.
