# Supplementary material for: Dietary Polyphenols as Regulators of Protein Deposition and Muscle Health: Mechanisms, Interactions, and Functional Applications
Source: Food Sci Nutr. 2026 Nov 1;14(11):e72487. doi: 10.1002/fsn3.72487 (PMC13724988; doi:10.1002/fsn3.72487)
Supplement: Supplementary file 1 [file FSN3-14-e72487-s001.docx]

**Dietary Polyphenols as Regulators of Protein Deposition and Muscle Health: Mechanisms, Interactions, and Functional Applications**

Chen Liu^1,2,3^, Mengyi Ren^2^, Xin Lai^1,4^, Ke Gu^1,5^, Liqiang Tan^2,3*^, Aimin Wu^1,*^, Daiwen Chen^1,*^

*^1^* Institute of Animal Nutrition, Sichuan Agricultural University, Chengdu, 611130, Sichuan, P. R. China

*^2^* College of Horticulture, Sichuan Agricultural University, Chengdu, 611130, Sichuan, P. R. China

*^3^* Sichuan Provincial Key Laboratory of Tea Resources Utilization and Quality Assessment, Chengdu, 611130, Sichuan, P. R. China

*^4^* Institute of Subtropical Agriculture, Chinese Academy of Sciences, Changsha, 410125, P. R. China

*^5^* Nutrition, Eumetabolism and Health Group, Girona Biomedical Research Institute (IDIBGI-CERCA), 17190 Salt, Girona, Spain

* Corresponding authors at: Institute of Animal Nutrition, Sichuan Agricultural University, Chengdu, 611130, Sichuan, P. R. China (D. Chen and A. Wu); College of Horticulture, Sichuan Agricultural University, Chengdu, 611130, Sichuan, P. R. China (L. Tan)

*E-mail addresses*: [tanliqiang@sicau.edu.cn](mailto:tanliqiang@sicau.edu.cn) (L. Tan), [wuaimin0608@163.com](mailto:wuaimin0608@163.com) (A. Wu), [dwchen@sicau.edu.cn](mailto:dwchen@sicau.edu.cn) (D. Chen

**Table S1.** In vitro cell models for studying the positive effects of dietary polyphenols on protein regulation and potential muscle health.

| **Cell type** | **Modeling agent/method** | **Polyphenol and dosage** | **Experimental duration** | **Effect** | **Key mechanism** | **Reference** |
| --- | --- | --- | --- | --- | --- | --- |
| C2C12 (mouse skeletal muscle precursor cell line) | 3D-clinorotation | EC, ECG, EGCG and Quercetin (125 μM) | 24 h | Inhibited  the expression of atrogin to prevent muscle atrophy | Inhibiting ERK phosphorylation downregulates the expression levels of atrogin-1 and MuRF-1 | (Hemdan et al., 2009) |
|  | TNF-α (50 ng/ml) | EGCG (10 and 50 μM) | 4 or 24 h | Alleviated TNF-α-induced suppression of protein synthesis | Inhibiting the PKR enzyme to attenuate TNF-α-induced protein synthesis suppression | (Mirza et al., 2014) |
|  | Serum deprivation | EGCG (10 μM) | 24 h | Slowed down protein degradation | Inhibiting the expression of proteasome 20S and 19S subunits, while suppressing MuRF1 and MAFbx, and increasing the expression of pAkt and pFoxO3a | (Mirza et al., 2014) |
|  | \ | Urolithin B (15 μM) | 24 h | Enhanced C2C12 myotube differentiation and inhibited protein degradation | Increasing the diameter of myotubes and reduced the mRNA levels of FoxO1, FoxO3, and the ubiquitin ligases MAFbx and MuRF1 | (Rodriguez et al., 2017) |
|  | \ | EC (20 μM) | 48 h | Promoted myogenic differentiation | Activating the p38 MAPK and Akt pathways, enhancing the expression of MHC, MyoD, and myogenin, and increasing MyoD activity as well as the heterodimerization of MyoD and E proteins | (Lee et al., 2017) |
|  | Prmt7/FNDC5 siRNA knockdown | Apigenin (2.5 or 5 μM) | 2 or 6 d | Promoted myogenic differentiation | Increasing protein expression of total MHC, MHC2A, and MHC2B, enhancing Prmt7 protein expression levels, and modulating the Prmt7-p38-myoD and Akt-S6K1 pathways | (Jang et al., 2017) |
|  | TNF-α (100 ng/mL) | Isoflavones (20 - 100 μM) | 24 h | Anti-inflammatory and inhibited myotube atrophy | Inhibiting MuRF1 expression, activating SIRT1 through the AMPK pathway, and suppressing NF-κB activation | (Hirasaka et al., 2013) |
|  | Dexamethasone (1 μM) | Glabridin (10 μM) | 24.5 h | Inhibited protein degradation in myotubes | Inhibiting the expression of MuRF1 and Cbl-b, as well as the phosphorylation of p38 and FoxO3a | (Yoshioka et al., 2019) |
|  | Dexamethasone (1 μM) | Quercetin (1 - 10 μM) | 28 h | Alleviated protein degradation | Enhancing phosphorylation of Akt downstream of the myostatin pathway and increasing expression of atrogenes | (Otsuka et al., 2019) |
|  | Dexamethasone (1 μM) | Delphinidin (1, 5 and 10 μM) | 24 h | Prevented protein degradation | Inhibiting dexamethasone -induced Cbl-b expression | (Murata et al., 2016) |
|  | Dexamethasone (1 μM) | Resveratrol (100 μM) | 24 h | Alleviated atrophy | Inhibiting NF-κB activation in C2C12 myotubes and blocking MuRF1 expression | (Sun et al., 2017) |
|  | Palmitate (250 μM) | Oligonol (10, 25, 50 μg/mL) | 24 h | Improved impaired myotube formation | Inhibiting Atrogin-1 and MuRF1 mRNA expression | (Liu et al., 2017) |
|  | TNF-α (100 ng/mL) | Quercetin (20 μM) | 48 h | Prevented protein degradation | Inhibiting the expression of muscle atrophy factors MAFbx/Atrogin-1 and MuRF1, and increasing HO-1 protein levels | (Kim et al., 2018) |
|  | CT26 conditioned medium | Canadine (100 nM) | 27 h | Inhibited muscle protein degradation and stimulated myogenesis | Downregulating the muscle-specific E3 ligases MAFbx/atrogin-1 and MuRF1 | (H. Lee et al., 2017) |
| L6 muscle cells (rat skeletal muscle myoblast cell line) | Dexamethasone (1 μM) | Resveratrol (100 μM) | 24 h | Inhibited protein degradation and alleviated muscle fiber atrophy | Suppressing atrogin-1 and MuRF1 expression in the muscle fibers | (Alamdari et al., 2012) |
|  | Palmitate acid (PA, 0.75 mM) | Resveratrol (25 μM) | 24 h | Alleviated PA-induced cell death and muscle fiber atrophy | Activating the PKA/LKB1/AMPK signaling pathway | (Huang et al., 2019) |
|  | Dexamethasone (2 μM) | Dihydromyricetin (10 μM) | 24 h | Reversed mitochondrial dysfunction, inhibited protein degradation, promoted protein synthesis | Reversing mitochondrial dysfunction through the PGC-1α/TFAM and PGC-1α/MFN2 signaling pathways, inactivating FoxO3a, and activating the AKT/mTOR pathway | (Huang et al., 2018) |
| SMA fibroblasts | \ | Caffeic acid (20 μM), Chlorogenic acid (50 μM), and Curcumin (20 μM) | 24 h | Alleviated proximal spinal muscular atrophy | Activating the promoter increased the levels of FL-SMN2 mRNA in SMA fibroblasts | (Dayangac-Erden et al., 2011) |
| Immortalized myotubes (Human skeletal myoblast line) | Oligomycin | Hesperetin (10 μM) | 48 and 72 h | Enhanced cellular antioxidant capacity and protein levels | Reducing ROS, upregulating Nrf2 and Pgc-1alpha protein levels | (Biesemann et al., 2018) |

**Table S2.** Animal models for studying the effects of dietary polyphenols on protein regulation and muscle health.

| **Animal** | **Modeling agent/method** | **Polyphenol and dosage** | **Experimental duration** | **Effect** | **Key mechanism** | **Reference** |
| --- | --- | --- | --- | --- | --- | --- |
| CD2F1 (BALB/c×DBA/2) F1 mice | Subcutaneous injection of C-26 adenocarcinoma cells, 150 μL | Resveratrol (100, 200 or 500 mg/kg) | Oral administration daily for 20 consecutive days | Prevented weight loss and inhibited cancer-induced skeletal muscle and cardiac muscle atrophy | Preventing tumor-induced reductions in lean body mass and fat mass in tumor-bearing mice, and inhibiting NF-κB transcription | (Shadfar et al., 2011) |
|  | Subcutaneous injection of C-26 adenocarcinoma cells, 150 μL | Quercetin (250 mg/kg) | Daily oral gavage for 21 consecutive days | Suppressed weight loss and muscle atrophy | Inhibiting the expression of MuRF1 and Atrogin-1 genes | (Velazquez et al., 2014) |
| BALB/c mice | Tail suspension | Diet containing 0.5% tea catechins | Daily oral administration for 24 consecutive days | Reduced skeletal muscle contractile dysfunction and mitigated muscle atrophy | Preventing the decline in muscle strength of the atrophied soleus muscle and reducing the levels of carbonyl myofibrillar proteins | (Ota et al., 2011) |
|  | Cisplatin (75 mg/m^2^/week, i.p.) | Magnolol (10 mg/kg/d) | Daily injections for 3 weeks | Inhibited muscle protein degradation and inflammatory responses, while promoted protein synthesis | Reducing the expression of myostatin, total FoxO3, MuRF1, and MAFbx proteins in muscle tissue | (Chen et al., 2015) |
| BALB/cCrSlc mice | Dexamethasone (0.001% w/v) | Quercetin glycosides (0.15% or 0.45% w/v) | Daily oral gavage for 21 consecutive days | Prevented Dexamethasone-induced muscle atrophy | Suppressing the mRNA expression levels of atrogin-1, MuRF-1, and myostatin | (Otsuka et al., 2019) |
| ICR mice | Denervation | Diet containing 0.5% (w/w) resveratrol | Daily feeding for 3 consecutive weeks | Attenuated muscle fiber atrophy | Preventing denervation-induced reductions in muscle weight and fiber atrophy, and inhibited atrogin-1 and p62 immunoreactivity | (Asami et al., 2018) |
| Sprague-Dawley (SD) rats | High-Fat Diet (HFD) | Diet containing 0.4% resveratrol | Daily administration for a duration of 20 weeks | Prevented muscle loss and improved mitochondrial morphological abnormalities | Enhancing grip strength and eliminating excessive fat accumulation, elevating the levels of PGC-1α, TFAM, and mfn2 proteins, while suppressing drp1 expression | (Huang et al., 2019) |
|  | Treadmill exercise | Resveratrol (150 mg/kg/d) | Daily administration for 6 consecutive weeks | Inhibited apoptosis and improved muscle mass | Increasing the expression of p-AMPK and SIRT1, reducing the acetylation of P53 and the Bax/Bcl-2 ratio, and activating the AMPK/Sirt1 pathway | (Liao et al., 2017) |
|  | d-Galactose (150 mg/kg/d,i.p.) | Ampelopsin (100 mg/kg/d) | Daily gavage administration for 6 consecutive weeks | Enhanced antioxidant and anti-inflammatory effects, inhibited apoptosis, and alleviated skeletal muscle atrophy | Reducing MDA levels in the gastrocnemius muscle, reversing the decrease in PGC-1α and irisin, preventing the reduction in the LC3-II/LC3-I ratio and Beclin1and inhibiting protein ubiquitination | (Kou et al., 2017) |
|  | Hypobaric hypoxia exposure | EGCG (200 mg/kg bw) | Daily oral administration for seven weeks | Attenuated protein degradation and alleviated muscle atrophy | Inhibiting the protein expression levels of the 19S and 20S proteasome subunits of myosin, as well as the ubiquitin ligases MuRF1 and MAFbx, while upregulating the expression levels of IL-15 and IGF-1 | (Meador et al., 2015) |
|  | Hypobaric hypoxia exposure | Curcumin (100  mg/kg bw) | Daily oral administration for 14 consecutive days | Inhibited muscle protein hydrolysis and increased muscle mass | Increasing the expression of myogenin, MyoD, and Myf5 in the gastrocnemius muscle, and elevating the content of slow skeletal muscle myosin heavy chain | (Chaudhary et al., 2019) |
|  | Dexamethasone (500 μg/kg bw) | Dihydromyricetin (100 or 200 mg/kg bw) | Daily intraperitoneal administration for 10 consecutive days | Preserved muscle mass and mitigated the loss of average fiber cross-sectional area, enhanced grip strength | Mitigating mitochondrial dysfunction: increasing the number of IFM, elevating levels of PGC-1α and TFAM, and suppressing the loss of mtDNA content | (Huang et al., 2018) |
| SAMP8 mice | HFD | HFD containing 0.5% GTEs | Fed three times daily for 4 consecutive months | Increased muscle mass and alleviated muscle atrophy | Stimulating AKT and S6K phosphorylation, preventing the reduction in muscle mass | (Onishi et al., 2018) |
| C57BL mice | \ | EC/EGCG (0.25% w/v) | Solution provided every other day for 37 to 44 weeks | Enhanced the survival rate of aged mice and delayed skeletal muscle degeneration | Counteracting the age-induced downregulation in the NAD pathway in both serum and skeletal muscle, as well as in the extracellular matrix and PPAR pathways within skeletal muscle | (Si et al., 2019) |
|  | \ | Oligonol (20 mg or 200 mg/kg in chow diet)) | Fed daily for 10 weeks | Alleviated muscle loss | Inhibiting Atrogin-1 and MuRF1 gene expression, reducing NF-κB expression in the nuclear fraction | (Liu et al., 2017) |
|  | HFD | HFD containing 0.05 % or 0.1% quercetin | Fed daily for 9 weeks | Reduced obesity-induced skeletal muscle atrophy | Reducing the levels of inflammatory cytokines/chemokines (TNF-α and MCP-1) in skeletal muscle, downregulating the transcriptional and protein levels of Atrogin-1 and MuRF1 | (Le et al., 2014) |
|  | Subcutaneous injection of LLC cells (3×10^5^ or 5×10^4^) | EGCG (0.2 or 0.6 mg) | Oral administration once daily for 32 or 34 days | Alleviated cancer cachexia-induced skeletal muscle atrophy | Inhibiting the mRNA expression of NF-κB, MuRF1, and MAFbx, modulating NF-κB-mediated ubiquitin-proteasome proteolysis | (Wang et al., 2011) |
|  | Subcutaneous injection of LLC cells (1×10^6^) | Isoflavones (0.4% w/w) | Oral gavage once daily for 3 weeks | Alleviated muscle atrophy | Inhibiting the expression of MAFbx/Atrogin-1 and MuRF1, as well as ERK phosphorylation | (Hirasaka et al., 2016) |
|  | \ | Modified AIN-76A diet with 0.2% and 0.4% apigenin | Daily feeding for 7 weeks | Promoted skeletal muscle hypertrophy and improved muscle function | Upregulating mRNA expression of MHC and GPR56 signaling, increasing Prmt7 expression, activating the mTORC1 pathway | (Jang et al., 2017) |
|  | Subcutaneous injection of Lewis lung cancer cells (200 µl, 1×10^7^) | Luteolin (20 mg/kg/d) | Oral gavage once daily for 24 days | Alleviated inflammation and reduced cancer-induced skeletal and cardiac muscle atrophy | Reducing the expression levels of MuRF1 and atrogin-1 in skeletal muscle, suppressing the inflammatory mediators TNF-α and IL-6, inhibiting the phosphorylation of IκB kinase β and p65 | (Chen et al., 2018) |
|  | Subcutaneous injection of LLC cells (1×10^5^) | Morin (0.1% w/w) | Daily feeding for 3 weeks | Prevented cancer cachexia-induced muscle atrophy | Inhibiting the reduction in muscle wet weight and fiber size | (Yoshimura et al., 2018) |
|  | Streptozocin (200 mg/kg bw, i.p.) | Diet containing 0.04% (w/w) resveratrol | Daily feeding for 8 weeks | Improved diabetes-Induced skeletal muscle atrophy | Improving muscle atrophy and muscle function, inhibiting the increase of MuRF-1, as well as LC3-II and cleaved caspase-3 in skeletal muscle | (Wang et al., 2018) |
|  | 5/6 nephrectomy | Resveratrol (200 mg/kg/d) | Daily gavage administration for 21 days | Increased muscle mass and alleviated muscle atrophy | Inhibiting MuRF1 mRNA levels and NF-κB phosphorylation | (Sun et al., 2017) |
|  | HFD | HFD containing 0.1% apigenin | Daily feeding for 8 weeks | Ameliorated obesity-induced skeletal muscle atrophy | Activating AMPK, reducing the expression of MuRF1 and Atrogin-1 genes | (Choi et al., 2017) |
|  | Sciatic nerve denervation | Diet containing 0.1% apigenin | Daily feeding for 2 weeks | Inhibited denervation-induced muscle atrophy | Reducing the expression of MuRF1, upregulating myosin heavy chain (MHC) and MHC type IIb, and suppressed the expression of TNF-α and IL-6 | (Choi et al., 2018) |
|  | HFD | HFD containing 0.05% quercetin | Daily feeding for 9 weeks | Inhibited obesity-induced skeletal muscle atrophy | Upregulating HO-1 protein expression in skeletal muscle, suppressing the expression of MuRF1 | (Kim et al., 2018) |
|  | HFD | Quercetin (0.05% or 0.1%) | Daily feeding for 9 weeks | Reduced obesity-induced skeletal muscle atrophy | Decreasing levels of TNF-α and MCP-1 in skeletal muscle, downregulating the transcription and protein levels of Atrogin-1 and MuRF1 | (Le et al., 2014) |
|  | Ankle fixation | 8-Prenylnaringenin (0.05%, 0.0005%, or 0.00005% by body weight) | Daily feeding for 20 days | Promoted the recovery from disuse muscle atrophy | Activating the phosphorylation of PI3K, 4E-BP1, Akt, and P70S6K1 | (Mukai, Horikawa, et al., 2016) |
|  | Denervation | Diet containing 0.2% quercetin | Daily feeding for 2 weeks | Prevented disuse muscle atrophy in denervated mice | Activating Akt phosphorylation, increasing plasma IGF-1 levels, upregulating mitochondrial biogenesis | (Mukai, Matsui, et al., 2016) |
|  | Treadmill endurance exercise | Hesperetin (50 mg/kg/d) | Orally administered once daily for 8 consecutive weeks | Alleviated muscle atrophy, improved overall muscle performance in aged mice | Enhancing mitochondrial energy supply, increasing the cross-sectional area of the gastrocnemius muscle | (Biesemann et al., 2018) |
|  | Dexamethasone (5 mg/kg bw/d) | Glabridin (40  mg/kg bw/d) | Orally administered once daily for 2 weeks | Inhibited muscle atrophy | Inhibiting the expression of MuRF1 and Cbl-b, the phosphorylation of FoxO3a, and the nuclear translocation of the glucocorticoid receptor | (Yoshioka et al., 2019) |
|  | Tail suspension | Delphinidin (4 or  20 mg/kg bw/d) | Orally administered once daily for 10 days | Inhibited muscle weight loss, prevented muscle atrophy | Inhibiting the expression of genes related to reactive oxygen species, ubiquitin ligases, and protein degradation | (Murata et al., 2016) |
|  | Hindlimb suspension | Flavan 3-ol (50 mg/kg /d) | Oral gavage administered daily for 14 consecutive days | Alleviated the atrophy of the gastrocnemius, tibialis anterior, and soleus muscles | Inhibiting the levels of ubiquitin ligases | (Ito et al., 2017) |
|  | Streptozocin (200 mg/kg bw, i.p.) | Curcumin (1500 mg /kg/d) | Daily feeding for 2 consecutive weeks | Inhibited protein degradation and ameliorated skeletal muscle atrophy | Inhibiting the gene expression of muscle-specific ubiquitin E3 ligases atrogin-1/MAFbx and MuRF1, suppressing inflammatory cytokines and oxidative stress | (Ono et al., 2015) |
|  | Unilateral sciatic nerve section | Urolithin B (10 μg/d) | Subcutaneous implantation of an osmotic pump for continuous delivery over 28 days | Reduced denervation-induced muscle weight loss, promoted skeletal muscle hypertrophy | Decreasing the mRNA levels of FoxO1, FoxO3, MAFbx, MuRF1, and myostatin, increasing the phosphorylation levels of mTOR and rpS6 in the tibialis anterior muscle | (Rodriguez et al., 2017) |
| Fisher 344 × Brown Norway rats | Hindlimb suspension | EGCG (50 mg/kg) | Oral gavage administered once daily for 3-5 weeks | Improved muscle recovery | Increasing BrdU-labeled nuclei in the soleus muscle, elevating phosphorylated Akt, reducing the expression of apoptosis-related proteins such as Bax and p53 | (Alway et al., 2014) |
|  | Hindlimb suspension | EGCG (50 mg/kg) | Oral gavage administered once daily for 3-5 weeks | Improved muscle health | Increasing the expression of autophagy-related genes (ATG16L2, SNCA, TM9SF1, Pink1, PIM-2), suppressing the protein abundance of Beclin1 and LC3-II/I | (Takahashi et al., 2017) |
|  | Hindlimb suspension | Resveratrol (125 mg/kg/d) | Oral gavage administered once daily for 3-5 weeks | Improved muscle quality after age-related atrophy | Reduced abundance of pro-apoptotic proteins Bax, cleaved caspase 3, and cleaved caspase 9 in the reloaded muscle | (Bennett et al., 2013) |
| Nude–nu male mice | Subcutaneous injection of MAC16 cells (200μl, 5×10⁶/ml) | Curcumin c3 complex (100 or 200 mg/kg bw) | Orally administered once daily for 20 days | Alleviated proteolysis and muscle atrophy | Inhibiting the expression of MURF-1/atrogin-1/MAFbx | (Siddiqui et al., 2009) |
| Zebrafish (wild type, AB line) | \ | Ferulic acid (0.2 mg/g) | Each tank is fed 500 mg of feed daily for 30 d | Promoted protein synthesis, increased muscle mass, and induced hypertrophic growth of fast skeletal muscle | Upregulating the mRNA expression levels of MyoD, myogenin, and SRF, along with their target genes encoding sarcomeric unit proteins involved in muscle hypertrophy, Increasing the phosphorylation levels of zTOR, p70S6K, and 4E-BP1 | (Wen & Ushio, 2017) |

**References**

Alamdari, N., Aversa, Z., Castillero, E., Gurav, A., Petkova, V., Tizio, S., & Hasselgren, P.-O. 2012. Resveratrol prevents dexamethasone-induced expression of the muscle atrophy-related ubiquitin ligases atrogin-1 and MuRF1 in cultured myotubes through a SIRT1-dependent mechanism. *Biochemical and Biophysical Research Communications*, *417*(1), 528-533. <https://doi.org/10.1016/j.bbrc.2011.11.154>

Alway, S. E., Bennett, B. T., Wilson, J. C., Edens, N. K., & Pereira, S. L. 2014. Epigallocatechin-3-gallate improves plantaris muscle recovery after disuse in aged rats. *Experimental Gerontology*, *50*, 82-94. <https://doi.org/10.1016/j.exger.2013.11.011>

Asami, Y., Aizawa, M., Kinoshita, M., Ishikawa, J., & Sakuma, K. 2018. Resveratrol attenuates denervation-induced muscle atrophy due to the blockade of atrogin-1 and p62 accumulation. *International Journal of Medical Sciences*, *15*(6), 628-637. <https://doi.org/10.7150/ijms.22723>

Bennett, B. T., Mohamed, J. S., & Alway, S. E. 2013. Effects of Resveratrol on the Recovery of Muscle Mass Following Disuse in the Plantaris Muscle of Aged Rats. *Plos One*, *8*(12), Article e83518. <https://doi.org/10.1371/journal.pone.0083518>

Biesemann, N., Ried, J. S., Ding-Pfennigdorff, D., Dietrich, A., Rudolph, C., Hahn, S., Hennerici, W., Asbrand, C., Leeuw, T., & Struebing, C. 2018. High throughput screening of mitochondrial bioenergetics in human differentiated myotubes identifies novel enhancers of muscle performance in aged mice. *Scientific Reports*, *8*, Article 9408. <https://doi.org/10.1038/s41598-018-27614-8>

Chaudhary, P., Sharma, Y. K., Sharma, S., Singh, S. N., & Suryakumar, G. 2019. High altitude mediated skeletal muscle atrophy: Protective role of curcumin. *Biochimie*, *156*, 138-147. <https://doi.org/10.1016/j.biochi.2018.10.012>

Chen, M.-C., Chen, Y.-L., Lee, C.-F., Hung, C.-H., & Chou, T.-C. 2015. Supplementation of Magnolol Attenuates Skeletal Muscle Atrophy in Bladder Cancer-Bearing Mice Undergoing Chemotherapy via Suppression of FoxO3 Activation and Induction of IGF-1. *Plos One*, *10*(11), Article e0143594. <https://doi.org/10.1371/journal.pone.0143594>

Chen, T., Li, B., Xu, Y., Meng, S., Wang, Y., & Jiang, Y. 2018. Luteolin reduces cancer-induced skeletal and cardiac muscle atrophy in a Lewis lung cancer mouse model. *Oncology Reports*, *40*(2), 1129-1137. <https://doi.org/10.3892/or.2018.6453>

Choi, W. H., Jang, Y. J., Son, H. J., Ahn, J., Jung, C. H., & Ha, T. Y. 2018. APIGENIN INHIBITS SCIATIC NERVE DENERVATION-INDUCED MUSCLE ATROPHY. *Muscle & Nerve*, *58*(2), 314-318. <https://doi.org/10.1002/mus.26133>

Choi, W. H., Son, H. J., Jang, Y. J., Ahn, J., Jung, C. H., & Ha, T. Y. 2017. Apigenin Ameliorates the Obesity-Induced Skeletal Muscle Atrophy by Attenuating Mitochondrial Dysfunction in the Muscle of Obese Mice. *Molecular Nutrition & Food Research*, *61*(12), Article 1700218. <https://doi.org/10.1002/mnfr.201700218>

Dayangac-Erden, D., Bora-Tatar, G., Dalkara, S., Demir, A. S., & Erdem-Yurter, H. 2011. Carboxylic acid derivatives of histone deacetylase inhibitors induce full length SMN2 transcripts: a promising target for spinal muscular atrophy therapeutics. *Archives of Medical Science*, *7*(2), 230-234. <https://doi.org/10.5114/aoms.2011.22072>

Hemdan, D. I. I., Hirasaka, K., Nakao, R., Kohno, S., Kagawa, S., Abe, T., Harada-Sukeno, A., Okumura, Y., Nakaya, Y., Terao, J., & Nikawa, T. 2009. Polyphenols prevent clinorotation-induced expression of atrogenes in mouse C2C12 skeletal myotubes [; Research Support, Non-U.S. Gov't]. *The journal of medical investigation : JMI*, *56*(1-2), 26-32. <https://doi.org/10.2152/jmi.56.26>.

Hirasaka, K., Maeda, T., Ikeda, C., Haruna, M., Kohno, S., Abe, T., Ochi, A., Mukai, R., Oarada, M., Teshima-Kondo, S., Ohno, A., Okumura, Y., Terao, J., & Nikawa, T. 2013. Isoflavones Derived from Soy Beans Prevent MuRF1-Mediated Muscle Atrophy in C2C12 Myotubes through SIRT1 Activation. *Journal of Nutritional Science and Vitaminology*, *59*(4), 317-324. <https://doi.org/10.3177/jnsv.59.317>.

Hirasaka, K., Saito, S., Yamaguchi, S., Miyazaki, R., Wang, Y., Haruna, M., Taniyama, S., Higashitani, A., Terao, J., Nikawa, T., & Tachibana, K. 2016. Dietary Supplementation with Isoflavones Prevents Muscle Wasting in Tumor-Bearing Mice. *Journal of Nutritional Science and Vitaminology*, *62*(3), 178-184. <https://doi.org/10.3177/jnsv.62.178>.

Huang, Y., Chen, K., Ren, Q., Yi, L., Zhu, J., Zhang, Q., & Mi, M. 2018. Dihydromyricetin Attenuates Dexamethasone-Induced Muscle Atrophy by Improving Mitochondrial Function via the PGC-1α Pathway. *Cellular Physiology and Biochemistry*, *49*(2), 758-779. <https://doi.org/10.1159/000493040>.

Huang, Y., Zhu, X., Chen, K., Lang, H., Zhang, Y., Hou, P., Ran, L., Zhou, M., Zheng, J., Yi, L., Mi, M., & Zhang, Q. 2019. Resveratrol prevents sarcopenic obesity by reversing mitochondrial dysfunction and oxidative stress via the PKA/LKB1/AMPK pathway. *Aging-Us*, *11*(8), 2217-2240. <https://doi.org/10.18632/aging.101910>.

Ito, M., Kudo, N., Miyake, Y., Imai, T., Unno, T., Yamashita, Y., Hirota, Y., Ashida, H., & Osakabe, N. 2017. Flavan 3-ol delays the progression of disuse atrophy induced by hindlimb suspension in mice. *Experimental Gerontology*, *98*, 120-123. <https://doi.org/10.1016/j.exger.2017.07.010>

Jang, Y. J., Son, H. J., Choi, Y. M., Ahn, J., Jung, C. H., & Ha, T. Y. 2017. Apigenin enhances skeletal muscle hypertrophy and myoblast differentiation by regulating Prmt7. *Oncotarget*, *8*(45), 78300-78311. <https://doi.org/10.18632/oncotarget.20962>

Kim, Y., Kim, C.-S., Joe, Y., Chung, H. T., Ha, T. Y., & Yu, R. 2018. Quercetin Reduces Tumor Necrosis Factor Alpha-Induced Muscle Atrophy by Upregulation of Heme Oxygenase-1. *Journal of Medicinal Food*, *21*(6), 551-559. <https://doi.org/10.1089/jmf.2017.4108>

Kou, X., Li, J., Liu, X., Yang, X., Fan, J., & Chen, N. 2017. Ampelopsin attenuates the atrophy of skeletal muscle from D-gal-induced aging rats through activating AMPK/SIRT1/PGC-1α signaling cascade. *Biomedicine & Pharmacotherapy*, *90*, 311-320. <https://doi.org/10.1016/j.biopha.2017.03.070>

Le, N. H., Kim, C.-S., Park, T., Park, J. H. Y., Sung, M.-K., Lee, D. G., Hong, S.-M., Choe, S.-Y., Goto, T., Kawada, T., & Yu, R. 2014. Quercetin Protects against Obesity-Induced Skeletal Muscle Inflammation and Atrophy. *Mediators of Inflammation*, *2014*, Article 834294. <https://doi.org/10.1155/2014/834294>

Lee, H., Lee, S.-J., Bae, G.-U., Baek, N.-I., & Ryu, J.-H. 2017. Canadine from *Corydalis turtschaninovii* Stimulates Myoblast Differentiation and Protects against Myotube Atrophy. *International Journal of Molecular Sciences*, *18*(12), Article 2748. <https://doi.org/10.3390/ijms18122748>

Lee, S.-J., Leem, Y.-E., Go, G.-Y., Choi, Y., Song, Y. J., Kim, I., Kim, D. Y., Kim, Y. K., Seo, D.-W., Kang, J.-S., & Bae, G.-U. 2017. Epicatechin elicits MyoD-dependent myoblast differentiation and myogenic conversion of fibroblasts. *Plos One*, *12*(4), Article e0175271. <https://doi.org/10.1371/journal.pone.0175271>

Liao, Z.-Y., Chen, J.-L., Xiao, M.-H., Sun, Y., Zhao, Y.-X., Pu, D., Lv, A.-K., Wang, M.-L., Zhou, J., Zhu, S.-Y., Zhao, K.-X., & Xiao, Q. 2017. The effect of exercise, resveratrol or their combination on Sarcopenia in aged rats via regulation of AMPK/Sirt1 pathway. *Experimental Gerontology*, *98*, 177-183. <https://doi.org/10.1016/j.exger.2017.08.032>

Liu, H.-W., Chen, Y.-J., Chang, Y.-C., & Chang, S.-J. 2017. Oligonol, a Low-Molecular Weight Polyphenol Derived from Lychee, Alleviates Muscle Loss in Diabetes by Suppressing Atrogin-1 and MuRF1. *Nutrients*, *9*(9), Article 1040. <https://doi.org/10.3390/nu9091040>

Meador, B. M., Mirza, K. A., Tian, M., Skelding, M. B., Reaves, L. A., Edens, N. K., Tisdale, M. J., & Pereira, S. L. 2015. The Green Tea Polyphenol Epigallocatechin-3-Gallate (EGCg) Attenuates Skeletal Muscle Atrophy in a Rat Model of Sarcopenia. *The Journal of frailty & aging*, *4*(4), 209-215. <https://doi.org/10.14283/jfa.2015.58>

Mirza, K. A., Pereira, S. L., Edens, N. K., & Tisdale, M. J. 2014. Attenuation of muscle wasting in murine C2C12 myotubes by epigallocatechin-3-gallate. *Journal of Cachexia Sarcopenia and Muscle*, *5*(4), 339-345. <https://doi.org/10.1007/s13539-014-0139-9>

Mukai, R., Horikawa, H., Lin, P.-Y., Tsukumo, N., Nikawa, T., Kawamura, T., Nemoto, H., & Terao, J. 2016. 8-Prenylnaringenin promotes recovery from immobilization-induced disuse muscle atrophy through activation of the Akt phosphorylation pathway in mice. *American Journal of Physiology-Regulatory Integrative and Comparative Physiology*, *311*(6), R1022-R1031. <https://doi.org/10.1152/ajpregu.00521.2015>

Mukai, R., Matsui, N., Fujikura, Y., Matsumoto, N., Hou, D.-X., Kanzaki, N., Shibata, H., Horikawa, M., Iwasa, K., Hirasaka, K., Nikawa, T., & Terao, J. 2016. Preventive effect of dietary quercetin on disuse muscle atrophy by targeting mitochondria in denervated mice. *Journal of Nutritional Biochemistry*, *31*, 67-76. <https://doi.org/10.1016/j.jnutbio.2016.02.001>

Murata, M., Kosaka, R., Kurihara, K., Yamashita, S., & Tachibana, H. 2016. Delphinidin prevents disuse muscle atrophy and reduces stress-related gene expression. *Bioscience Biotechnology and Biochemistry*, *80*(8), 1636-1640. <https://doi.org/10.1080/09168451.2016.1184560>

Onishi, S., Ishino, M., Kitazawa, H., Yoto, A., Shimba, Y., Mochizuki, Y., Unno, K., Meguro, S., Tokimitsu, I., & Miura, S. 2018. Green tea extracts ameliorate high-fat diet induced muscle atrophy in senescence-accelerated mouse prone-8 mice. *Plos One*, *13*(4), Article e0195753. <https://doi.org/10.1371/journal.pone.0195753>

Ono, T., Takada, S., Kinugawa, S., & Tsutsui, H. 2015. Curcumin ameliorates skeletal muscle atrophy in type 1 diabetic mice by inhibiting protein ubiquitination. *Experimental Physiology*, *100*(9), 1052-1063. <https://doi.org/10.1113/ep085049>

Ota, N., Soga, S., Haramizu, S., Yokoi, Y., Hase, T., & Murase, T. 2011. Tea catechins prevent contractile dysfunction in unloaded murine soleus muscle: A pilot study. *Nutrition*, *27*(9), 955-959. <https://doi.org/10.1016/j.nut.2010.10.008>

Otsuka, Y., Egawa, K., Kanzaki, N., Izumo, T., Rogi, T., & Shibata, H. 2019. Quercetin glycosides prevent dexamethasone-induced muscle atrophy in mice. *Biochemistry and biophysics reports*, *18*, 100618-100618. <https://doi.org/10.1016/j.bbrep.2019.100618>

Rodriguez, J., Pierre, N., Naslain, D., Bontemps, F., Ferreira, D., Priem, F., Deldicque, L., & Francaux, M. 2017. Urolithin B, a newly identified regulator of skeletal muscle mass. *Journal of Cachexia Sarcopenia and Muscle*, *8*(4), 583-597. <https://doi.org/10.1002/jcsm.12190>

Shadfar, S., Couch, M. E., McKinney, K. A., Weinstein, L. J., Yin, X., Rodriguez, J. E., Guttridge, D. C., & Willis, M. 2011. Oral Resveratrol Therapy Inhibits Cancer-Induced Skeletal Muscle and Cardiac Atrophy In Vivo. *Nutrition and Cancer-an International Journal*, *63*(5), 749-762. <https://doi.org/10.1080/01635581.2011.563032>

Si, H., Wang, X., Zhang, L., Parnell, L. D., Ahmed, B., LeRoith, T., Ansah, T.-A., Zhang, L., Li, J., Ordovas, J. M., Si, H., Liu, D., & Lai, C.-Q. 2019. Dietary epicatechin improves survival and delays skeletal muscle degeneration in aged mice (vol 33, pg 965, 2019) [Correction]. *Faseb Journal*, *33*(5), 6683-6683. <https://doi.org/10.1096/fj.201801163RERR>

Siddiqui, R. A., Hassan, S., Harvey, K. A., Rasool, T., Das, T., Mukerji, P., & DeMichele, S. 2009. Attenuation of proteolysis and muscle wasting by curcumin c3 complex in MAC16 colon tumour-bearing mice. *British Journal of Nutrition*, *102*(7), 967-975. <https://doi.org/10.1017/s0007114509345250>

Sun, L.-J., Sun, Y.-N., Chen, S.-J., Liu, S., & Jiang, G.-R. 2017. Resveratrol attenuates skeletal muscle atrophy induced by chronic kidney disease via MuRF1 signaling pathway. *Biochemical and Biophysical Research Communications*, *487*(1), 83-89. <https://doi.org/10.1016/j.bbrc.2017.04.022>

Takahashi, H., Suzuki, Y., Mohamed, J. S., Gotoh, T., Pereira, S. L., & Alway, S. E. 2017. Epigallocatechin-3-gallate increases autophagy signaling in resting and unloaded plantaris muscles but selectively suppresses autophagy protein abundance in reloaded muscles of aged rats. *Experimental Gerontology*, *92*, 56-66. <https://doi.org/10.1016/j.exger.2017.02.075>

Velazquez, K. T., Enos, R. T., Narsale, A. A., Puppa, M. J., Davis, J. M., Murphy, E. A., & Carson, J. A. 2014. Quercetin Supplementation Attenuates the Progression of Cancer Cachexia in Apc<SUP>Min/+</SUP> Mice. *Journal of Nutrition*, *144*(6), 868-875. <https://doi.org/10.3945/jn.113.188367>

Wang, D., Sun, H., Song, G., Yang, Y., Zou, X., Ha, P., & Li, S. 2018. Resveratrol Improves Muscle Atrophy by Modulating Mitochondrial Quality Control in STZ-Induced Diabetic Mice. *Molecular Nutrition & Food Research*, *62*(9), Article 1700941. <https://doi.org/10.1002/mnfr.201700941>

Wang, H., Lai, Y.-J., Chan, Y.-L., Li, T.-L., & Wu, C.-J. 2011. Epigallocatechin-3-gallate effectively attenuates skeletal muscle atrophy caused by cancer cachexia. *Cancer Letters*, *305*(1), 40-49. <https://doi.org/10.1016/j.canlet.2011.02.023>

Wen, Y., & Ushio, H. 2017. Ferulic Acid Promotes Hypertrophic Growth of Fast Skeletal Muscle in Zebrafish Model. *Nutrients*, *9*(10), Article 1066. <https://doi.org/10.3390/nu9101066>

Yoshimura, T., Saitoh, K., Sun, L., Wang, Y., Taniyama, S., Yamaguchi, K., Uchida, T., Ohkubo, T., Higashitani, A., Nikawa, T., Tachibana, K., & Hirasaka, K. 2018. Morin suppresses cachexia-induced muscle wasting by binding to ribosomal protein S10 in carcinoma cells. *Biochemical and Biophysical Research Communications*, *506*(4), 773-779. <https://doi.org/10.1016/j.bbrc.2018.10.184>

Yoshioka, Y., Kubota, Y., Samukawa, Y., Yamashita, Y., & Ashida, H. 2019. Glabridin inhibits dexamethasone-induced muscle atrophy. *Archives of Biochemistry and Biophysics*, *664*, 157-166. <https://doi.org/10.1016/j.abb.2019.02.006>
